# Potential for hydrogen-oxidizing chemolithoautotrophic and diazotrophic populations to initiate biofilm formation in oligotrophic, deep terrestrial subsurface waters

**DOI:** 10.1186/s40168-017-0253-y

**Published:** 2017-03-23

**Authors:** Xiaofen Wu, Karsten Pedersen, Johanna Edlund, Lena Eriksson, Mats Åström, Anders F. Andersson, Stefan Bertilsson, Mark Dopson

**Affiliations:** 1 Centre for Ecology and Evolution in Microbial Model Systems (EEMiS), Linnaeus University, 391 82 Kalmar, Sweden; 2 Microbial Analytics Sweden AB, Mölnlycke, Sweden; 3 Department of Biology and Environmental Science, Linnaeus University, Kalmar, Sweden; 4 KTH Royal Institute of Technology, Science for Life Laboratory, School of Biotechnology, Stockholm, Sweden; 5 Department of Ecology and Genetics, Limnology and Science for Life Laboratory, Uppsala University, Uppsala, Sweden

**Keywords:** 16S rRNA gene, Metagenome, Binning, Terrestrial, Deep biosphere, Biofilm formation, Metabolism

## Abstract

**Background:**

Deep terrestrial biosphere waters are separated from the light-driven surface by the time required to percolate to the subsurface. Despite biofilms being the dominant form of microbial life in many natural environments, they have received little attention in the oligotrophic and anaerobic waters found in deep bedrock fractures. This study is the first to use community DNA sequencing to describe biofilm formation under in situ conditions in the deep terrestrial biosphere.

**Results:**

In this study, flow cells were attached to boreholes containing either “modern marine” or “old saline” waters of different origin and degree of isolation from the light-driven surface of the earth. Using 16S rRNA gene sequencing, we showed that planktonic and attached populations were dissimilar while gene frequencies in the metagenomes suggested that hydrogen-fed, carbon dioxide- and nitrogen-fixing populations were responsible for biofilm formation across the two aquifers. Metagenome analyses further suggested that only a subset of the populations were able to attach and produce an extracellular polysaccharide matrix. Initial biofilm formation is thus likely to be mediated by a few bacterial populations which were similar to *Epsilonproteobacteria*, *Deltaproteobacteria*, *Betaproteobacteria*, *Verrucomicrobia*, and unclassified bacteria.

**Conclusions:**

Populations potentially capable of attaching to a surface and to produce extracellular polysaccharide matrix for attachment were identified in the terrestrial deep biosphere. Our results suggest that the biofilm populations were taxonomically distinct from the planktonic community and were enriched in populations with a chemolithoautotrophic and diazotrophic metabolism coupling hydrogen oxidation to energy conservation under oligotrophic conditions.

**Electronic supplementary material:**

The online version of this article (doi:10.1186/s40168-017-0253-y) contains supplementary material, which is available to authorized users.

## Background

Microbial life in natural environments typically occurs in biofilms adhered to surfaces via a matrix of extracellular polymeric substances (EPS). This is despite the higher energetic costs compared to the free-living state, e.g., up-regulation of genes involved in motility during attachment [1]. Biofilm formation involves a number of sequential steps. These include an establishment phase characterized by motility, cell-to-cell communication, initial adhesion, and EPS production [2, 3]. Living in a biofilm can confer many ecological advantages including more efficient nutrient recycling [4], genetic exchange [5], resistance to grazing [6], stress tolerance [7], and facilitation of syntrophy plus metabolite exchange [8].

The deep biosphere is separated from the light-driven surface by the time needed for waters to penetrate to these depths and is also set apart by other environmental conditions such as high pressure, stable reducing conditions, extensive exposure to mineral surfaces, and increasing temperatures with depth. The deep biome is the largest microbial ecosystem on earth [9], and the deep continental biosphere is estimated to host 2 to 19% of earth’s total biomass [10]. Although a large proportion of the earth’s microbial cells reside in the deep biosphere, our knowledge of the biology and identity of these organisms is scarce [11]. One reason for this is the difficulty to obtain uncontaminated samples. The present study was carried out at the Äspö Hard Rock Laboratory (Äspö HRL). This 460-m-deep underground laboratory circumvents many problems associated with contamination of the low biomass samples of the deep biosphere [11]. These advantages include that the groundwaters are physically separated from the oxidizing environment in the tunnel and that the flow of water into the boreholes is by gravity rather than pumping.

Intrusive igneous rocks make up the vast majority of the earth’s crust. These rocks are typically considerably fractured, providing space for water transport and habitats for microbial life. Despite being highly oligotrophic, at least a portion of the microbes in fracture groundwaters are proposed to be active. This is consistent with the presence of up to ~10 μM concentrations of hydrogen [12] suggested to support lithotrophic growth [13, 14] and the occurrence of bacteriophages that depend on active microorganisms to survive [15, 16]. Most knowledge of the terrestrial deep biosphere is from studies of free-living cells, revealing communities comprised of anaerobes capable of reducing nitrate, ferric iron, and sulfur or sulfate alongside methanogens and acetogens. All of these energy conservation strategies are supported by carbon dioxide and hydrogen as carbon and energy sources [12, 17–21]. Recent metagenomic studies also point to extensive metabolic versatility with heterotrophic, mixotrophic, and autotrophic metabolic strategies [22, 23]. However, as a substantial fraction of the microbiome in the deep biosphere is likely to live in biofilms [24, 25], the earlier work may provide an incomplete and possibly misleading description of this biome.

Despite biofilms in rock fractures being present in the deep biosphere [24–26], the factors controlling biofilm formation are poorly understood, especially in deep subsurface environments. This study is the first study to use metagenomic sequencing to address this knowledge gap.

## Results and discussion

### Geochemical characteristics of the borehole waters

Boreholes KA2198A (300 m below sea level) and KF0069A01 (450 m below sea level) are connected to water-bearing fissures in the bedrock (Additional file 1: Figure S1). Both borehole waters were near neutral (pH 7–8), contained dissolved sulfide (HS^−^), carried iron as Fe^2+^, and had stable chemistry and δ^18^O values over the measurement period extending from 2002 to 2008/2009 (Table 1 and Additional file 2: Figure S2). The observation that all available iron was reduced and that hydrogen sulfide was present suggested the waters were anaerobic. The presence of sulfate that acts as an electron acceptor in both waters supports that these groundwaters were substrate-limited and likely feature slow cellular growth rates.

**Table 1:** Chemical and isotopic composition of the groundwaters. Concentrations of chemical elements and ions are given in mg L^−1^ except for Cs which is in μg L^−1^. The ^18^O/^16^O ratio (δ^18^O) is reported via the δ notation “per mil” as a deviation from the Standard Mean Ocean Water (SMOW)

	KA2198A^a^		KF0069A01^b^	
	Med	Min-max	Med	Min-max
pH	7.5	7.4–7.5	7.8	7.2–7.8
Na	1465	1430–1530	3085	3040–3130
Mg	138	121–148	32	31–32
K	40	32–42	14	13–16
Ca	189	186–269	3870	3760–3980
Cl^−^	2780	2683–3050	13400	10810–14600
SO_4_^2−^	280	280–337	673	637–703
DOC	6.4	6.4–6.4	bd^c^	bd–bd
HCO_3_^−^	212	202–220	6.5	5.5–6.8
δ^18^O	−7.5	−7.8 to −7.4	−12.4	−12.4 to −12.4
Fe-total	1.7	1.5–1.7	0.037	0.011–0.063
Fe^2+^	1.6	1.2–1.7	0.06	db–0.06
Mn	0.71	0.70–0.97	0.23	0.23–0.23
HS^−^	0.05	0.01–0.08	0.01	bd–0.07
NH_4_^+^_N	2.4	2.3–3.1	0.01	0.01–0.02
PO_4_^3−^_P	0.0071	0.0006–0.0136	bd	bd–bd
NO_3_^−^	bd	bd–bd	bd	bd–bd
NO_2_^−^	0.00055	0.0005–0.0006	bd	bd–bd
Cs	4.6	4.5–4.8	4.2	4.2–4.2

^a^1–8 measurements in 2002–2009

^b^1–15 measurements in 2003–2008

^c^bd, below detection limit

KA2198A had high magnesium and potassium concentrations, which are tracers of marine waters [27], and also had slightly lower values for chloride and δ^18^O compared to modern brackish Baltic Sea water [28] (Table 1). This implies that this groundwater mainly consisted of infiltrated brackish Baltic Sea water, to some extent diluted with meteoric water, and was classified as “modern marine” (sample name defined as “MM”). The precise infiltration age of the groundwater was <20 years and probably even more recent. The groundwater cesium concentrations (4.5–4.8 μg L^−1^) were much higher than in surface waters in the region [29], indicating that the trace-metal hydrochemistry had been altered during and after the marine water infiltration [30]. The latest chemical measurements of this groundwater (from 2009) predate the microbiological experiments (Additional file 2: Figure S2). However, the chemical characteristics were unlikely to have changed as, e.g., the chloride and sulfate concentrations in this type of groundwater within the Äspö HRL have not altered over time scales of decades [28]. KF0069A01 had typically high chloride concentrations and relatively low δ^18^O values for saline groundwater with a hydrological residence time in the order of millions of years [31] (Table 1). This groundwater was thus termed “old saline” (defined as “OS”). Its high concentrations of calcium, cesium, and sulfate result from mineral weathering and dissolution over millions of years [29], and the water also contains relatively low amounts of dissolved organic carbon, bicarbonate, and ammonium. This type of groundwater within the Äspö HRL has had overall stable chloride concentrations since the early 21st century [28], and therefore, the geochemical measurements of this groundwater (2003–2008) can be considered representative of the prevailing geochemistry in the borehole during the microbiological experiments.

### Sampling and sequencing data

Flow cells were directly connected to boreholes and the groundwater was allowed to pass under in situ temperature and pressure for 33 days. This time was sufficient to allow investigation of initial biofilm formation. The flow cells were loaded with garnet grains and glass beads as solid support for biofilm growth. These surfaces were chosen as (i) they were sterile, DNA-free, and RNAse/DNAse-free and could be sterilized by heating to 450 °C, respectively; (ii) in testing biofilm formation at the Äspö HRL, it was found that bedrock from the same environment was porous and that unattached minerals and particles disrupted DNA extractions from the formed biofilms [13, 14, 32]; and (iii) the fracture surfaces in the bedrock are mineralogically very heterogeneous consisting of various proportions of primary minerals (e.g., quartz, feldspar, plagioclase, and mica) and secondary precipitates (e.g., calcite, pyrite, epidote, Fe-oxides, and clay minerals) [33, 34]. Therefore, the flow cell system was simplified by using a single silicate mineral (garnet) that in terms of biofilm formation can be considered as representative for the silicate rocks (granites) in the Äspö HRL. However, the drawback of these solid supports was that they have different characteristics to the rock surfaces that potentially affected the initial biofilm forming populations.

The volume of groundwater that passed through the flow cells and details of the 16S rRNA gene sequencing data are given in Additional file 3: Table S1. The Illumina sequencing yielded 1.41 × 10^8^ to 1.70 × 10^8^ raw reads and 1.38 × 10^8^ to 1.65 × 10^8^ trimmed reads per sample that assembled into 5671 to 48857 contigs ≥1000 bp in length (Additional file 4: Table S2). The contigs were binned into 33 garnet and 35 glass near-complete metagenome-assembled genomes (MAGs) from the modern marine water and 11 garnet and 9 glass MAGs from the old saline, representing 44.4 ± 5.3% of the reads. The MAGs contained ≥31 of the 36 CONCOCT single-copy genes, with an estimated bin completeness of ≥86% (Additional file 5: Table S3).

### Estimation of biofilm and planktonic cell numbers

Based upon normalized ATP measurements for the different conditions in the two contrasting groundwaters, the modern marine water flow cells had consistently higher estimated cell abundances with 4.6 × 10^6^ and 2.0 × 10^8^ cells cm^2^ on the garnet (sample name defined as “MMR”) and glass surfaces (defined as “MMG”), respectively. This compared to 1.9 × 10^4^ and 4.3 × 10^3^ cells cm^2^ on the corresponding solid surfaces from the old saline water (defined as “OSR” and “OSG”; Additional file 6: Table S4). The higher cell abundance in the biofilms of the modern marine water was consistent with the greater DOC in this water type (Table 1). For comparison, calculated planktonic cell concentrations for the modern marine borehole was 2.8 × 10^4^ cells mL^−1^ [35]. The planktonic cell concentration was not measured for the old saline water, but water from an adjacent borehole with similar chemical characteristics was estimated to hold approximately 100 cells mL^−1^ [22]. Hence, the biofilms were quantitatively significant components of these deep aquifer systems and should be considered if we are to understand the interplay between the biosphere and geosphere.

Even with the extremely oligotrophic conditions in the Äspö HRL fracture waters, earlier data from cell growth in response to addition of putative substrates such as hydrogen and carbon dioxide, suggests that deep biosphere microorganisms are viable [13, 14, 36]. In the present study, direct measurements of ATP showed that the investigated microbial communities were metabolically active (Additional file 6: Table S4). This is further corroborated by the cells attaching to and colonizing solid surfaces within a 33-day period without any experimental manipulation of resources and with respective numbers of cells per square centimeter in the modern marine garnet and glass biofilms being ~164 and ~7000 fold greater than the number of planktonic cells in a milliliter of water.

### Microbial diversity from 16S rRNA gene sequencing and metagenome binning

The rarefaction curves of 16S rRNA amplicons suggested that the sequencing depth was sufficient to describe the communities in all samples (Additional file 7: Figure S3). The most abundant OTUs from the garnet and glass surface biofilms in both water types were affiliated to the genus *Sulfurimonas* (relative abundance of 48.7 to 68.2%; Table 2). However, all the abundant OTUs (≥1% abundance) were almost completely distinct between the planktonic and biofilm habitats in the respective water types (Table 2). This could be due to preferential growth of some taxa in biofilms [37] or alternatively, the result of planktonic samples being collected a year prior to sampling for biofilms. The second alternative was deemed unlikely as previous experience from Äspö HRL and other underground research laboratories suggests that microbial communities in deep groundwaters remain stable over longer periods [38, 39]. Both the species richness (Chao1 and ACE) and diversity (Shannon-Weaver and Inverse Simpson) were lower in the modern marine biofilms compared to the free-living planktonic cells (Additional file 8: Table S5). In contrast, the richness was lower in the old saline groundwater compared to these biofilms while the estimated diversity decreased. This was due to an increase in low abundance populations (<0.1%) in the old saline biofilm compared to the planktonic fractions (Additional file 8: Table S5). This decrease in diversity with separation from the light-driven surface was similar to that observed in planktonic populations at the Äspö HRL [40].

**Table 2:** Relative abundance (%) of OTUs in bacterial 16S rRNA gene v4v6 sequence libraries. Sequences with ≥1% abundance frequency are shown in detail. The data are from three PCR-generated amplicon samples that were pooled before sequencing

	Relative abundance (%)					
OTUs	MM planktonic	MM garnet	MM glass	OS planktonic	OS garnet	OS glass
Unknown phylum	29.4	7.51	13.0	50.0	2.54	3.05
*Actinobacteria* ^a^	-	-	-	1.62	-	-
*Atribacteria* ^a^	1.18	-	-	20.6	-	-
*Gracilibacteria* ^a^	5.30	9.85	2.16	-	-	-
*Gammaproteobacteria* ^b^	12.3	7.89	4.44	-	-	-
*Thermoplasmata* ^b^	-	-	-	2.75	-	-
*Desulfuromonadales* ^c^	-	-	-	-	-	1.21
*Ignavibacteriales* ^c^	-	-	-	8.13	-	-
*Myxococcales* ^c^	-	-	-	-	1.29	1.37
*Hydrogenophilaceae* ^d^	-	-	1.30	-	-	-
*Rhodocyclaceae* ^d^	-	1.57	-	-	15.7	27.6
*Desulfatiglans* ^e^	3.30	-	-	-	-	-
*Desulfobulbus* ^e^	-	3.22	-	-	-	-
*Desulfocapsa* ^e^	-	-	-	-	5.33	8.74
*Ferritrophicum* ^e^	-	3.07	1.32	-	-	-
*Marichromatium* ^e^	1.86	-	-	-	-	-
*Sideroxydans* ^e^	-	-	2.95	-	-	-
*Sulfurimonas* ^e^	-	48.7	50.9	-	68.2	50.6
*Sulfurovum* ^e^	-	3.78	8.41	-	-	-
*Syntrophus* ^e^	3.78	-	-	2.40	-	-
Candidate division OP3^f^	12.6	-	3.21	-	-	-
Candidate Nitrotoga^f^	-	4.59	4.50	-	-	1.11
Omnitrophica^f^	-	-	-	1.15	-	-
Parcubacteria^f^	11.7	-	-	7.54	-	-
TA06^f^	-	-	1.22	-	-	-
<1%	18.6	9.80	6.58	5.78	6.92	6.26

Classification: ^a^phylum, ^b^class, ^c^order, ^d^family, ^e^genus, and ^f^candidate division

Abbreviations: *MM* modern marine, *OS* old saline

**Fig. 1:**
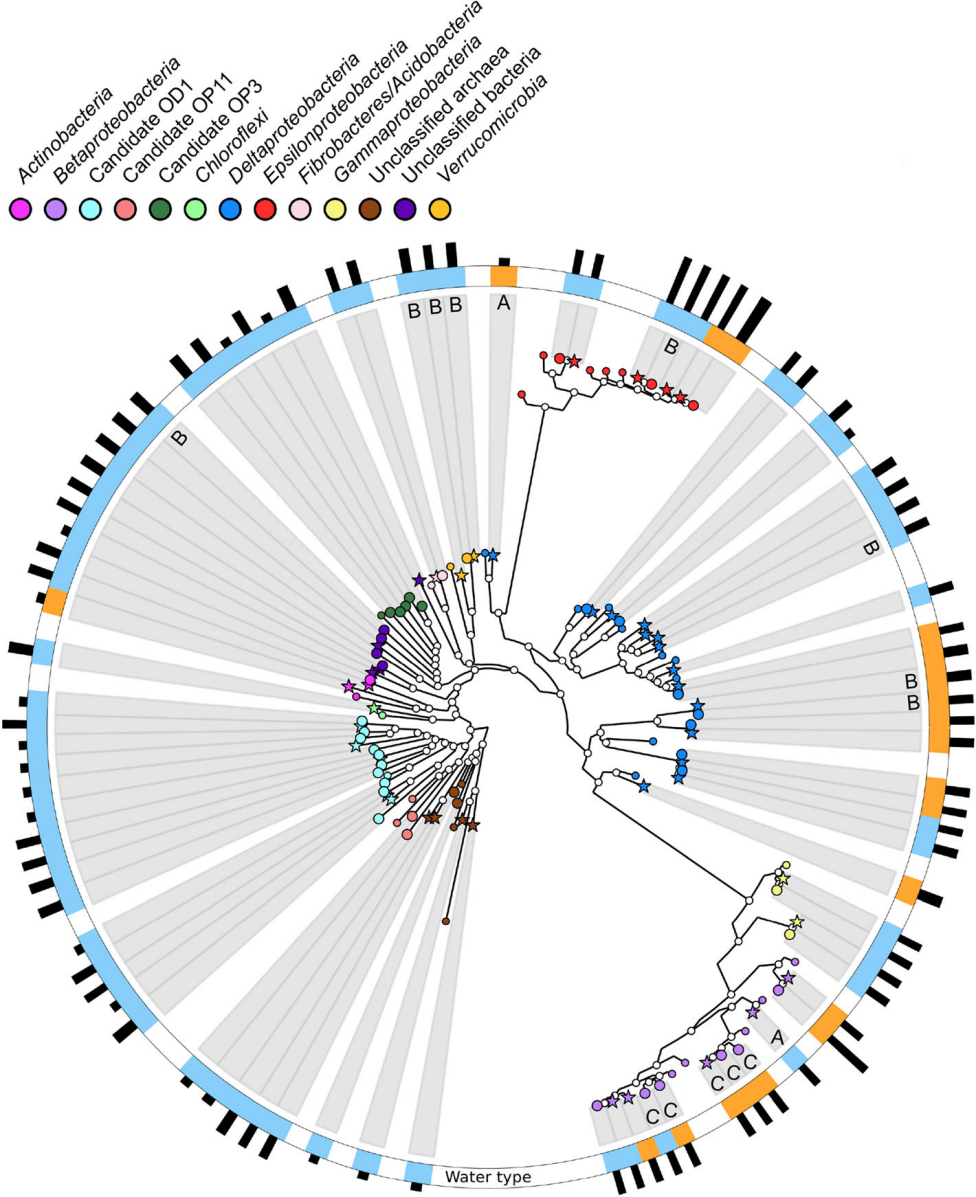
Phylogenetic tree showing alignment of the MAGs to reference sequences. Marker genes were extracted from the metagenome bins and aligned to sequenced genomes from isolates or to assembled genomes for lineages lacking a representative isolate (marked with small colored circles; see Additional file 9: Figure S4 for details). The clades marked with *stars* and *large circles* correspond to bins obtained from biofilm grown on garnets and glass, respectively. The first ring shows the water type with the color-coding: modern marine (*blue*) and old saline (*yellow*). The second ring indicates the relative community abundance based upon percentage mapped reads with the shortest line corresponding to <0.1% of the population, the medium length line for 0.1 to 10% of the population, and the longest line representing >10% of the population. *A*, *B*, and *C* represent the groups able to form a biofilm in Fig. 3

Inspecting the most abundant populations represented by near-complete genomes, three MAGs (totaling 29.8% of the reads) from the modern marine water garnet biofilms were assigned to the *Epsilonproteobacteria* compared to two MAGs from the glass surface biofilms (totaling 15.7% of the reads; Fig. 1; Additional file 5: Table S3 and Additional file 9: Figure S4). Of these *Epsilonproteobacteria* MAGs, two and three were affiliated with the sulfate reducing genera *Sulfurovum* and *Sulfurimonas*, respectively. In the old saline water, members of *Sulfurimonas* were also abundant, making up 32.9 and 24.8% of the total reads in the metagenomes from the garnet and glass biofilms, respectively. Sulfate-reducing MAGs were also detected in the biofilms including one most similar to *Desulfovibrio aespoeensis* in the old saline water garnet biofilm, but this population was not seen on the glass surface. Several other MAGs with the capacity to reduce sulfate were observed and seemed to be distinct for the respective water masses (Additional file 9: Figure S4 and Additional file 10: Figure S5). The modern marine garnet and glass metagenomes also contained MAGs related to the ferrous iron-oxidizing *Betaproteobacteria* species *Siderooxydans lithotrophicus* (1.9 and 5.7%, respectively). In addition, the modern marine biofilms contained MAGs that affiliated with candidate phyla, totaling 3.0 and 2.6% of the reads. Finally, four (1.1%) and two (0.2%) archaeal MAGs were identified in the modern marine garnet and glass metagenomes, respectively.

The biofilm MAGs were compared with planktonic cell MAGs from borehole groundwaters in the Äspö HRL [22]. This suggested MMG_Bin_14 (Candidatus OD1) was similar to a population present in borehole SA1229A also containing modern marine water. In addition, OSR_Bin_45, OSG_Bin_23, and OSG_Bin_24 aligning with *Thiobacillus denitrificans* were similar to a planktonic population from old saline water borehole KA3385A:1. The suggested overlap of just two MAG populations between planktonic and biofilm populations from separate studies supported the observation that these communities were dissimilar. In addition, the identified community was different from an established biofilm previously recovered from an in situ rock surface [26], suggesting that the biofilm community would further develop as it matures, such as by the inclusion of species incapable of initiating the biofilm formation.

### Partition of genes between biofilm and planktonic populations

**Fig. 2:**
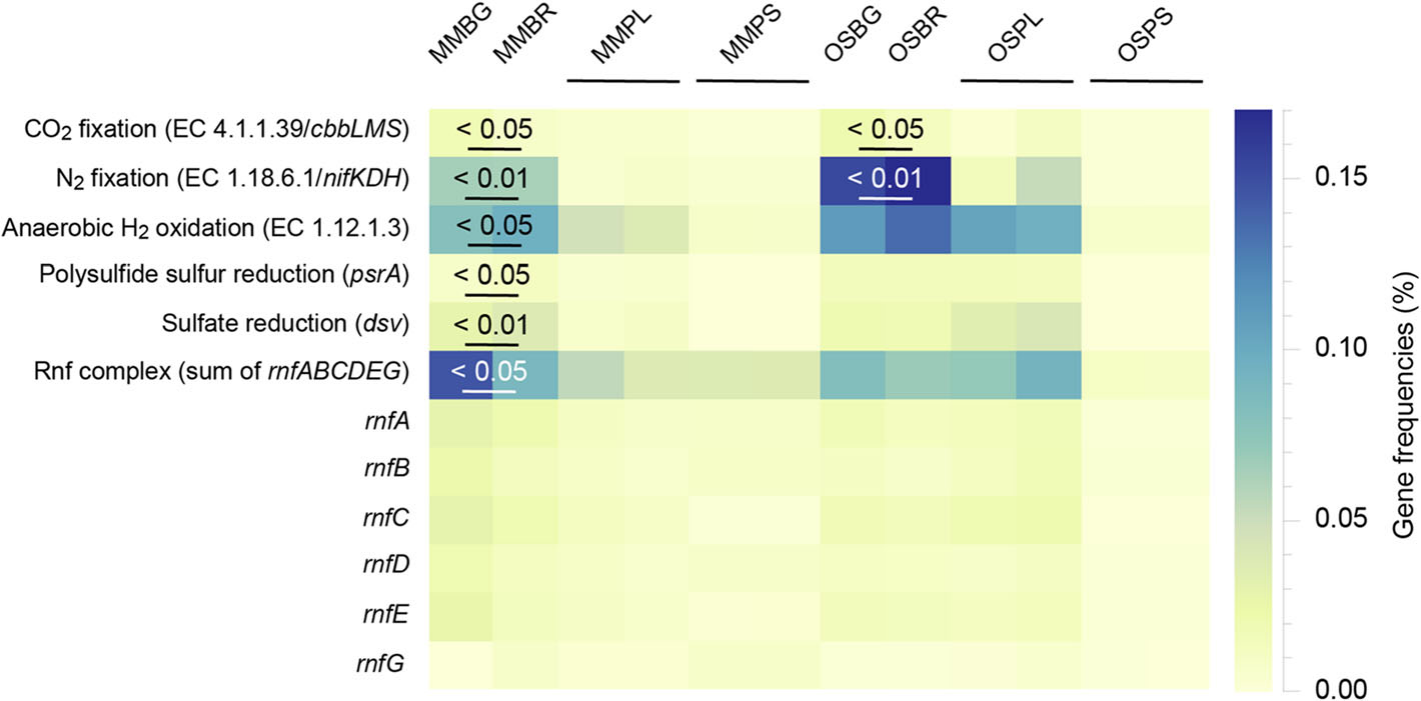
Heat map of gene frequencies for selected characteristics in the biofilm and planktonic populations. The figure shows percentage gene frequencies for modern marine biofilms formed on glass (MMBG) and garnet (MMBR) compared to modern marine planktonic large (MMPL) and small (MMPS) cells and old saline biofilms formed on glass (OSBG) and garnet (OSBR) compared to duplicate metagenomes for both the old saline planktonic large (OSPL) and small (OSPS) cells. The frequencies for CO_2_ fixation are based upon the presence of the *cbbLMS* genes coding for the Enzyme Commission number (EC) 4.1.1.39; nitrogen fixation is for EC 1.18.6.1 encompassing the *nifKDH* genes; and sulfate reduction is solely based upon *dsv* as no *dsrAB* genes were identified. In addition, frequencies are shown for the Rnf complex as well as for the individual genes. Statistics are presented when the difference between biofilm values for garnet and glass (number of replicates =1 each) compared to large and small planktonic cells (number of replicates =2 each) have *p* values <0.05 or <0.01 (*p* values were not calculated for the individual Rnf genes)

An earlier meta-analysis of gene frequencies from metagenome studies revealed positive correlations with actual rates of metabolic processes in the respective environments [41]. Gene frequencies in biofilms (this study) compared with planktonic populations [22] in boreholes fed by the same water types suggested strong partitioning of functions between the respective communities (Fig. 2 plus Additional file 11: Table S6 and Additional file 12: Figure S6). Genes coding for carbon dioxide fixation (based upon the presence of the *cbbLMS* genes coding for the Enzyme Commission number (EC) 4.1.1.39) had greater representation in the modern marine and old saline biofilm communities compared to the small and large cell planktonic populations (one-way ANOVA; *F* = 9.6, *p* < 0.05 and *F* = 8.2, *p* < 0.05 for the sum of the *cbbLMS* genes, respectively; Additional file 11: Table S6). This trend was also true for nitrogen fixation gene frequencies (EC 1.18.6.1 with at least two of the *nifKDH* genes) in the modern marine and old saline biofilm communities (*F* = 2146.6, *p* < 0.01 and *F* = 57.9, *p* < 0.01 for the sum of the *nifKDH* genes, respectively). Genes predicted to be involved in anaerobic hydrogen oxidation (EC 1.12.1.3) were also more highly represented in the modern marine biofilm compared to planktonic cells (*F* = 15.8, *p* = < 0.05), while there was no statistically significant difference for this trait between old saline water biofilm and planktonic communities. Genes encoding the use of polysulfide sulfur (*psrA*; *F* = 11.1, *p* < 0.05) and sulfate (based solely on the *dsv* gene as no *dsrAB* genes were identified; *F* = 38.6, *p* < 0.01) as terminal electron acceptors were more abundant in the modern marine water biofilm, suggesting dominance of electron transport rather than fermentation. The Rnf complex (at least four of *rnfABCDEG*) had higher frequencies in modern marine biofilms (*F* = 16.5, *p* < 0.05 for the sum of the *rnfABCDEG* genes) and combined with the widespread occurrence of these genes across both water types, we propose that this respiration mechanism may constitute a generic adaptation to oligotrophy in the waters at the Äspö HRL. All of these data were consistent with prevailing oligotrophic conditions necessitating a chemolithoautotrophic and diazotrophic metabolism that couples hydrogen oxidation with electron transport [12] for acquisition of nutrients and energy for biofilm formation.

### Potential for biofilm formation

The flow cells were run for a total of 33 days and generation times for attached microbial populations in the studied deep hard rock fractures have been extrapolated from cell counts to be between 16 and 90 days [14, 42]. Therefore, the cells attached to the solid surfaces had likely not proliferated more than at most one generation and largely represent cells imported from the aqueous phase. This experimental design allowed the investigation of populations with the genetic potential to initiate biofilm formation prior to the attachment of later colonizing populations that lack such traits.

**Fig. 3:**
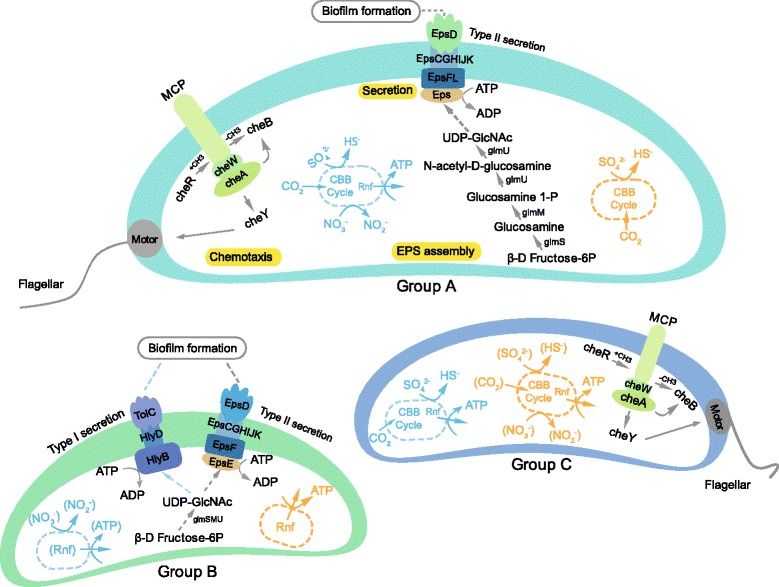
Model of biofilm formation derived from gene assignments from the MAGs. Group A is suggested to have chemotaxis, motility using a flagellum, and production of EPS that is subsequently transported outside of the cell. Group B represents MAGs suggested to be able to produce and secrete EPS. Group C shows the MAGs encoding genes suggested to provide motility. *Grey arrows* indicate that the path was present in both modern marine water and old saline water, and *blue arrows* in Group B means only modern marine water bins had type I secretion system

The MAGs were searched for genes implicated in biofilm formation. This search included genes involved in chemotaxis (presence of methyl-accepting chemotaxis protein (MCP) and all *cheABWRY* genes) which provides an advantage during biofilm formation [43]; genes for flagella (presence of at least 20 of *flgLKDGHIBCE, fliDCEFGMNHIOPQR*, *flhAB*, and both *motAB* genes for the flagellum motor), which aid in bacterial attachment [43, 44]; and genes to produce EPS (*galU* or both *galUE*) and export these polymers to the cell surface (*hlyBD* plus *tolC* for Type I secretion or *eps* for Sec-dependent secretion), a trait that provides mechanical stability and facilitates adhesion to the surface [45] (Table 3 and Additional file 13: Table S7). Based upon these criteria, potential surface colonizers for the two water types were assigned to three groups (A to C; Fig. 3 and Additional file 13: Table S7). No complete pathways for quorum sensing were identified. However, *luxS* homologs that are linked to production of autoinducer-2 signals in *Epsilonproteobacteria* [46] were detected in all MAGs affiliated with this class.

**Table 3:** Summary of the metabolic pathways for the reconstructed genomes. The table provides gene homologs for chemotaxis, motility, and EPS production and secretion for biofilm formation

Group	MAGs	Biofilm formation	Metabolic pathways and energy conservation	Nutrient fixation	% mapped reads^a^
Modern marine water					
A	MMR_Bin_58	EPS production, secretion and motility	H_2_ oxidation, sulfate and nitrate reduction and Rnf	CO_2_	0.13
B	MMR_Bins_36, 41 and 98/MMG_Bins_22, 48 and 93	EPS production and secretion	(Fermentation)^b^, (formate and H_2_ oxidation), (Sulfur reduction), (nitrate reduction), (denitrification) and (Rnf)	(N_2_)	1.18 13.36
C	MMG_Bin_17	Motility	Formate and H_2_ oxidation, sulfate/sulfur reduction and Rnf	CO_2_ and N_2_	3.10
Old saline water					
A	OSR_Bin_1	EPS production, secretion and motility	Formate oxidation, Sulfate sulfur reduction	CO_2_ and N_2_	0.22
B	OSR_Bin_39/ OSG_Bin_9	EPS production and secretion	Fermentation, H_2_ oxidation, Rnf	None	0.65 0.34
C	OSR_Bin_45/ OSG_Bins_16, 23 and 24	Motility	(Formate oxidation)	(CO_2_) and (N_2_)	1.71 2.82

^a^Total percentage of mapped reads of all bins within the group

^b^Brackets designates that not all populations in the group contain genes suggested for the trait

In the modern marine water, only one MAG (MMR_Bin_58; assigned to Group A) featured all traits for biofilm formation capacity (i.e., chemotaxis, flagellum-assisted motility, and EPS production and export). This MAG represented 0.13% of the mapped reads from modern marine water garnet surface biofilm. In contrast, none of the MAGs from the glass biofilm contained all these traits for surface attachment and biofilm formation. This may have been due to syntrophic interactions between populations able to e.g., attach to the surface allowing subsequent populations that lack this ability to attach and build a viable biofilm.

The second group of potential biofilm-producing populations (able to produce EPS and transport it to the cell surface) included MMR_Bin_58 was most closely related to *Sulfuricella denitrificans*, a facultative anaerobe that couples sulfur oxidation with nitrate reduction [47]. However, the metagenome assembly also suggests that MMR_Bin_58 grows via anaerobic hydrogen oxidation coupled to sulfate reduction, potentially producing an ion motive force via the ferredoxin:NAD^+^ oxidoreductase Rnf complex and fixing carbon dioxide via the Calvin-Benson-Bassham (CBB) cycle. Three MAGs from the modern marine water garnet surface (totaling 1.2% of the mapped reads) and three MAGs from the modern marine water glass surface (13.4% of the mapped reads) contained genes implicated in EPS production and export (assigned to Group B). MMR_Bin_36, MMR_Bin_41, and MMG_Bin_48 were affiliated with the *Verrucomicrobia* and MMG_Bin_22 was from candidate division OP3. All of these MAGs were suggested to generate an ion motive force via the Rnf complex. In addition, MMR_Bin_41 and MMG_Bin_48 also had the potential to utilize hydrogen via type III anaerobic hydrogen oxidation. MMR_Bin_98 (assigned to a poorly defined clade within the *Deltaproteobacteria*) and MMG_Bin_93 (most related to *Sulfurimonas denitrificans*) were suggested to have the potential to oxidize formate (*fdh* gene), convert nitrate to nitrite or nitrogen gas (*nar/nap* and *nar*/*nir/nor/nos* genes, respectively), and fix nitrogen for cellular growth. In modern marine water, only one MAG (MMG_Bin_17; 3.10% of the mapped reads) was suggested to be able to sense chemical stimuli and move via a flagellum and was defined as Group C. This MAG contained genes assigned to hydrogen and formate oxidation, sulfate and sulfur reduction, carbon dioxide and nitrogen fixation, and the Rnf complex. Although the modern marine water was ostensibly anaerobic, both MMG_Bin_17 and OSG_Bin_16 were most closely related to *Sideroxydans lithotrophicus*, an iron-oxidizing bacterium that grows at oxic-anoxic interfaces [48].

The old saline water contained one MAG (OSR_Bin_1; 0.22% of the reads) that was suggested to code for all genes required for biofilm formation (group A). This population affiliated most closely to *D. aespoeensis* [49] and was suggested to reduce sulfate to sulfide during anaerobic respiration. However, OSR_Bin_1 also contained gene homologs suggested to encode enzymes involved in formate oxidation, carbon dioxide fixation via the CBB cycle, and nitrogen assimilation. The old saline water biofilms also contained OSR_Bin_39 and OSG_Bin_9 from Group B (0.65% and 0.34% of mapped reads, respectively) that contain genes to produce and export EPS. These bins were potentially able to metabolize pyruvate to either acetate or ethanol (all genes in one of the eight fermentation pathways to acetate or two out of three genes for the three pyruvate fermentation pathways to ethanol), oxidize hydrogen, and generate an ion motive force via the Rnf complex. Group C, suggested to sense chemical stimuli and move via a flagellum, was represented by one MAG on the garnet surface of the old saline water (OSR_Bin_45; 1.7% of reads) and three MAGs on the glass surfaces (OSG_Bin_16, OSG_Bin_23 and OSG_Bin_24; totaling 2.8% of reads). OSR_Bin_45 and OSG_Bin_24 from the old saline water were both phylogenetically similar to *T. denitrificans* [50]. These MAGs contained genes assigned to carbon dioxide fixation by the CBB cycle, nitrogen assimilation, formate oxidation to carbon dioxide, sulfate and nitrate reduction, and generation of an ion motive force via the Rnf complex. OSG_Bin_16 showed the potential ability to reduce sulfate to sulfide, fix carbon dioxide and nitrogen, and generate a proton motive force via the Rnf complex.

### Potential metabolism in deep biosphere biofilms

All MAGs were scrutinized for genes encoding electron donor and acceptor use as well as carbon dioxide and nitrogen fixation (Table 3 and Additional file13: Table S7). There were no major differences in the predicted metabolic pathways for MAGs implicated in initial biofilm formation between either the water types or surfaces.

The metabolic potential of the surface attached communities’ implied organic carbon (e.g., MMR_Bin_67 and OSG_Bin_4) and/or hydrogen oxidation (e.g., MMG_Bin_17 and OSR_Bin_28) coupled to reduction of sulfur or sulfate for energy acquisition. Additional populations were suggested to link organic carbon (e.g., MMG_Bin_16 and OSR_Bin_36) and/or hydrogen oxidation (MMR_Bin_58 and OSR_Bin_6) to nitrate reduction. Both of these observations are in agreement with previous studies from the Äspö HRL [22, 51]. The dominant populations in both the modern marine and old saline water biofilm formers were putative carbon- and nitrogen-fixers (MMG_Bin_93 representing 11.73% of the reads and OSG_Bin_24 representing 2.15% of the reads). The importance of previously unknown microorganisms with a simple, fermentative growth strategy has recently been recognized at the Äspö HRL [22] as well as at another site [20]. These earlier findings are consistent with the metabolic features of several biofilm populations identified here (e.g., MMR_Bin_23 and OSR_Bin_0). Hence, the biofilm community seems able to utilize a range of carbon and energy sources.

## Conclusions

Populations predicted to carry out all stages of biofilm formation in the deep terrestrial biosphere were taxonomically distinct from the planktonic community. The biofilm contained a mixed community dominated by hydrogen-fed autotrophs able to fix nitrogen that reflected the oligotrophic conditions in the waters of deep terrestrial aquifers.

## Methods

### Description of the Äspö HRL site

The Swedish Nuclear Fuel and Waste Management Company (SKB) operated Äspö HRL is situated on the southeastern coast of Sweden (Lat N 57° 26′ 4′′ Lon E 16° 39′ 36′′) in a bedrock dominated by 1800 Ma granite and quartz monzodiorite [12, 52]. The structure of the Äspö HRL tunnel has been shown in a previous study [53], and the geology, chemistry, and hydrology of the boreholes extending from the tunnel have been described [28, 54, 55]. The boreholes were sampled for planktonic cells, and flow cells attached to investigate for biofilm development (described below).

### Chemical and isotopic composition of the water

Descriptions of the analytical techniques and precision of the variables are given elsewhere: Cl^−^ and δ^18^O [28]; Na, K, Ca, Mg, Cs, and NH_4_^+^ [29]; Fe^2+^, Fe-total, dissolved organic carbon (DOC), and HS^−^ [56]; and Mn, SO_4_^2−^, HCO_3_^−^, and PO_4_^3−^ [30]. Multiple measurements were taken over a maximum of 7 years with median plus minimum/maximum values presented.

### Biofilm growth, sampling, DNA preparation, and sequencing

The flow cells were directly attached to the boreholes in the Äspö HRL tunnel. They had a stainless steel shell (length 300 mm, diameter 65 mm), were lined with polyvinyldifloride plastic, and were equipped with manometers and a pressure relief valve to enable biofilms to form under the high in situ pressure and low redox conditions prevailing in the fissures intersected by the boreholes (Additional file 1: Figure S1). Each flow cell had a 120-mm-long polyvinyldifloride insert with a 22 × 32 mm opening that supported ~100 g of sterile, DNA-free and RNAse/DNAse-free garnet grains (0.7 mm in diameter; MOBIO Laboratories (USA)) and glass beads (1 mm in diameter; VWR International). Glass beads were sterilized by heating to 450 °C for 5 h in a muffle furnace. The flow cells were connected to boreholes KA2198A and KF0069A01 from 23 May 2013 until 24 June 2013 (33 days). Immediately after disconnection from the borehole, the capped flow cells were transported at 4 °C to the laboratory (transport time <8 h), and DNA extraction was carried out on the same day. Approximately, 6 g of each support material was collected from the flow cells and used for DNA preparation.

Planktonic cells from the boreholes were collected on 2 February 2012 for KA2198A and 20 July 2012 for KF0069A01 by filtration under pressure through 47-mm diameter and 0.22-μm pore size membrane filters (supplied with the PowerWater® DNA Isolation Kit, (MO BIO Laboratories, Immuno diagnostics, Hämeenlinna, Finland) contained within a stainless steel filter holder (Millipore) at a flow rate of 0.2 L min^−1^ for 16 and 16.5 h, respectively. The filters were aseptically removed, placed in sample tubes provided with the PowerWater Kit, and stored frozen at −20 °C until DNA extraction. Genomic DNA from groundwater filters plus garnet grains and glass beads was extracted using the PowerWater Kit according to the protocol provided by the manufacturer.

A Bacterial 16S rDNA v4v6 amplicon library for sequencing was generated by using the degenerative forward (518F, CCAGCAGCYGCGGTAAN) [57] and reverse primer (1064R, CGACRRCCATGCANCACCT) [58]. Conditions for the PCR reaction were 1× Platinum HiFi Taq polymerase buffer, 1.6 units Platinum HiFi polymerase, 3.7 mM MgSO_4_, 200 μM dNTPs (PurePeak polymerization mix, ThermoFisher), and 400 nM primers. Between 5 and 25 ng of sample DNA was added to a master mix to a final volume of 100 μL, and this was divided into three replicate 33-μL reactions. Cycling conditions included an initial denaturation at 94 °C for 3 min; 30 cycles of 94 °C for 30 s, 57–60 °C for 45 s, and 72 °C for 1 min; and a final extension at 72 °C for 2 min using a Bio-Rad mycycler. The quality and concentration of the amplicon library was evaluated by using the Agilent Tapestation 2000 instrument according to the manufacturer’s protocol. The reactions were cleaned and products under 300 bp were removed using AMpure beads at 0.75 × volume (Beckman Coulter, Brea CA). The final products were re-suspended in 100 μL of 10 mM Tris-EDTA + 0.05% Tween-20, quantified using PicoGreen Quant-IT assay (Life Technologies), and assayed once again on the Tapestation 2000 instrument. Amplicons were further titrated in equimolar concentration before emulsion-PCR based on their dsDNA concentrations. A GS-FLX Sequencer was used to generate pyrotag sequence reads with the Roche Titanium reagents.

Metagenome libraries of extracted DNA from garnet grains and glass beads were prepared using the ThruPlex DNA-seq Kit with 96 dual indexes (Rubicon Genomics, MI, USA) using an Agilent NGS workstation (Agilent, CA, USA) and purified [59, 60]. The libraries were sequenced on an Illumina HiSeq (2 × 150 bp) in rapid mode at the Science for Life Laboratory in Stockholm, Sweden.

### Bioinformatic analysis

The 16S rRNA gene amplicon sequencing data was first trimmed to remove primer bases, barcodes, and low quality sequences [61]. The trimmed sequences were screened for chimeras by using the UCHIME algorithm [62]. Clustering into operational taxonomic units (OTUs) was by an open reference OTU-picking methodology with the USEARCH algorithm which uses both de novo and reference-based approaches [63]. Representative sequences were chosen from each OTU that were phylogenetically classified against the SILVA database (SILVA123_QIIME-release). Rarefaction to the lowest number of sequences (*n* = 10 000) was used to normalize sample count before analysis of the datasets.

Metagenome analysis was carried out as previously described [22]. In brief, adapters were removed with Seqprep before the sequences were trimmed and assembled using Sickle [64], Ray (version 2.3.1) [65], and Newbler (version 2.6). The assembled contigs were then binned to individual near-complete genomes using CONCOCT (version 0.3.0) [66]. CONCOCT uses 36 single-copy genes to evaluate the coverage of the assembled genome bins. The bins which had ≥31 single-copy genes and ≤2 duplicated single-copy genes were chosen for individual taxonomic and functional annotation using Phylosift v1.0. [67] and Prokka v1.10 [68]. Phylosift uses a suite of 37 marker genes for phylogenetic classification that are available at https://phylosift.wordpress.com/tutorials/scripts-markers/ [67]. Gene frequency comparisons between planktonic and biofilm samples were calculated by an in-house pipeline. In brief, all trimmed reads were first co-assembled using MEGAHIT v1.0.3 [69] with a minimum kmer size of 31 and a maximum kmer size of 81 at a step of 10. The assembled contigs (of ≥1000 bp in length) were then annotated using Prokka v1.10 [68]. Coverage (% of a locus represented in the assembly) of each gene predicted and annotated by Prokka was calculated using bedtools v2.17.0 [70] and the scripts prokkagff2bed.sh and get_coverage_for_genes.py, after the raw reads were mapped back to the assembly using the script map-bowtie2-markduplicates.sh. These scripts, developed by the Environmental Genomics group at SciLifeLab Stockholm, are available at http://metagenomics-workshop.readthedocs.io/. Gene coverage was calculated and normalized by dividing the coverage values by the total coverage of the sample. Frequencies of the genes of interest were computed and normalized by dividing the coverage values by the total coverage of the sample. The one-way ANOVA values were then calculated on the genes used to define the presence or absence of a pathway in the MAGs by averaging the normalized values and dividing the coverage values by the total coverage of the sample. The one-way ANOVAs were based on the biofilm values for garnet and glass (number of replicates =1 each) compared to large and small planktonic cells (number of replicates =2 each).

### ATP measurement and calculation of cell numbers

ATP was measured from the cells using the ATP Biomass Kit HS (BioThema, Sweden). The garnet grains and glass beads were added to 1 mL of reagent BS, vortexed for 30 s, and placed in the dark at room temperature for 30 min. Thereafter, the ATP was analyzed as previously described [71]. After analysis, the garnets and glass beads were washed, dried, and weighed for calculation of their total surface area. The total sampled surface area ranged from 8.5 to 15.0 cm^2^. The number of cells was then divided by the total area of the respective surface to get the approximate number of cells per cm^2^. ATP measurements were carried out in triplicate and averages ± standard deviation are presented.

## Additional files

Additional file 1: Figure S1. A flow cell connected to a borehole in the Äspö HRL tunnel. (PDF 195 kb)

Additional file 2: Figure S2. Geochemical measurements over time showing the stability of the two groundwater systems. (PDF 877 kb)

Additional file 3: Table S1. Samples from groundwater with the corresponding flow cell biofilm samples. The table shows decreasing diversity by depth below sea level (mbsl). Amounts of extracted double-stranded bacterial DNA analyzed fluorometrically using the Stratagene MX3005p fluorometer with MXPro software and the Quant-it Picogreen reagent kit from Molecular Probes. (PDF 13 kb)

Additional file 4: Table S2. Sequencing information for the four metagenomes. (PDF 60 kb)

Additional file 5: Table S3. Sequencing information for each approved phylogenetic bin from the metagenomes. (PDF 95 kb)

Additional file 6: Table S4. Extracted ATP and estimated cell numbers from the planktonic cells in the water phase as well as from the biofilms formed on garnet grains and glass beads. Values from this study presented as averages of three replicates ± SD. (PDF 78 kb)

Additional file 7: Figure S3. Rarefaction curves for bacterial 16S rRNA gene v4v6 dataset. Each curve represents a single sample and sampling occasion. (PDF 422 kb)

Additional file 8: Table S5. Species richness estimates (Chao1 and ACE) and diversity indices (Shannon-Weaver and Inverse Simpson) for the 16S rRNA gene sequencing. The >0.1 and >1% abundance taxa number was generated at genus level or the highest annotated rank. (PDF 14 kb)

Additional file 9: Figure S4. Whole-genome phylogenetic tree of the relationship between the CONCOCT bins visualized by Archaeopteryx. Scale bar equals 1.0%. (PDF 180 kb)

Additional file 10: Figure S5. Dendrogram of alignment from all near-complete reconstructed genomes (clustered bins showing >50% of the aligned base is the same). (PDF 578 kb)

Additional file 11: Table S6. Gene frequencies for selected characteristics in the modern marine (MM) and old saline waters (OS). Abbreviations: PL, planktonic large cells; PS, planktonic small cells; B, biofilm. (PDF 77 kb)

Additional file 12: Figure S6. Gene frequencies for selected characteristics (as defined in Table S6) in the modern marine (A) and old saline waters (B). Color coding: large (>0.22 μm) planktonic cells (black), small (<0.22 μm) planktonic cells (red), and biofilm cells (blue). Error bars denote standard deviations of duplicate samples. Abbreviation: ISC, inorganic sulfur compound. (PDF 405 kb)

Additional file 13: Table S7. Metabolic characteristics identified in the metagenomic bins from the two water types. The listed pathways are based upon BioCyc (http://biocyc.org/) and KEGG (http://www.genome.jp/kegg/). Additional pathways that were searched for but are not listed as they were negative in all cases include ferric reduction as a terminal electron acceptor; methanogenesis; aerobic and anaerobic ammonia oxidation; the reductive TCA cycle, incomplete TCA cycle, 3-hydroxypropanoate cycle, and reductive acetyl CoA pathway for CO_2_ fixation; and lipopolysaccharide production and export, type I and IV pili, autolysin gene *atlE* for release of extracellular DNA; and quorum sensing by acyl homoserine lactones and peptides. (PDF 100 kb)

## Abbreviations

Äspö HRL: Äspö Hard Rock Laboratory
CBB: Calvin-Benson-Bassham
DOC: Dissolved organic carbon
EPS: Extracellular polymeric substances
MAG: Metagenome-assembled genome

## References

[1] Kalmokoff M, Lanthier P, Tremblay TL, Foss M, Lau PC, Sanders G, Austin J, Kelly J, Szymanski CM (2006). Proteomic analysis of *Campylobacter jejuni* 11168 biofilms reveals a role for the motility complex in biofilm formation. J Bacteriol.

[2] Hori K, Matsumoto S (2010). Bacterial adhesion: from mechanism to control. Biochem Engin J.

[3] Petrova OE, Sauer K (2012). Sticky situations: key components that control bacterial surface attachment. J Bacteriol.

[4] van Gestel J, Vlamakis H, Kolter R. Division of labor in biofilms: the ecology of cell differentiation. Microbiol Spectrum. 2015;3:MB-0002-2014. 10.1128/microbiolspec.MB-0002-2014 26104716

[5] Merod RT, Wuertz S (2014). Extracellular polymeric substance architecture influences natural genetic transformation of *Acinetobacter baylyi* in biofilms. Appl Environ Microbiol.

[6] Sun SY, Tay QXM, Kjelleberg S, Rice SA, McDougald D (2015). Quorum sensing-regulated chitin metabolism provides grazing resistance to *Vibrio cholerae* biofilms. ISME J.

[7] Periasamy S, Nair HAS, Lee KWK, Ong J, Goh JQJ, Kjelleberg S, Rice SA. *Pseudomonas aeruginosa* PA01 exopolysaccharides are important for mixed species biofilm community development and stress tolerance. Front Microbiol. 2015;6: doi:10.3389/fmicb.2015.00851. 10.3389/fmicb.2015.00851 PMC4542536 26347731

[8] Morris BE, Henneberger R, Huber H, Moissl-Eichinger C (2013). Microbial syntrophy: interaction for the common good. FEMS Microbiol Rev.

[9] Edwards KJ, Becker K, Colwell F. The deep, dark energy biosphere: Intraterrestrial life on earth. In *Annu Rev Earth Planet Sci. Volume* 40. Edited by Jeanloz R. Palo Alto; 2012: 551-568

[10] McMahon S, Parnell J (2014). Weighing the deep continental biosphere. FEMS Microbiol Ecol.

[11] Wilkins MJ, Daly RA, Mouser PJ, Trexler R, Sharma S, Cole DR, Wrighton KC, Biddle JF, Denis EH, Fredrickson JK (2014). Trends and future challenges in sampling the deep terrestrial biosphere. Front Microbiol.

[12] Hallbeck L, Pedersen K (2008). Characterization of microbial processes in deep aquifers of the Fennoscandian Shield. Appl Geochem.

[13] Pedersen K (2012). Influence of H_2_ and O_2_ on sulphate-reducing activity of a subterranean community and the coupled response in redox potential. FEMS Microbiol Ecol.

[14] Pedersen K (2012). Subterranean microbial populations metabolize hydrogen and acetate under in situ conditions in granitic groundwater at 450 m depth in the Äspö Hard Rock Laboratory, Sweden. FEMS Microbiol Ecol.

[15] Eydal HS, Jagevall S, Hermansson M, Pedersen K (2009). Bacteriophage lytic to *Desulfovibrio aespoeensis* isolated from deep groundwater. ISME J.

[16] Kyle JE, Eydal HS, Ferris FG, Pedersen K (2008). Viruses in granitic groundwater from 69 to 450 m depth of the Äspö hard rock laboratory, Sweden. ISME J.

[17] Pedersen K (2013). Metabolic activity of subterranean microbial communities in deep granitic groundwater supplemented with methane and H_2_. ISME J.

[18] Itavaara M, Nyyssonen M, Kapanen A, Nousiainen A, Ahonen L, Kukkonen I (2011). Characterization of bacterial diversity to a depth of 1500 m in the Outokumpu deep borehole, Fennoscandian Shield. FEMS Microbiol Ecol.

[19] Nyyssonen M, Hultman J, Ahonen L, Kukkonen I, Paulin L, Laine P, Itavaara M, Auvinen P (2014). Taxonomically and functionally diverse microbial communities in deep crystalline rocks of the Fennoscandian Shield. ISME J.

[20] Purkamo L, Bomberg M, Nyyssonen M, Kukkonen I, Ahonen L, Itavaara M (2015). Heterotrophic communities supplied by ancient organic carbon predominate in deep Fennoscandian bedrock fluids. Microb Ecol.

[21] Dzaugis ME, Spivack AJ, Dunlea AG, Murray RW, D'Hondt S. Radiolytic hydrogen production in the subseafloor basaltic aquifer. Front Microbiol. 2016;7. 10.3389/fmicb.2016.00076. 10.3389/fmicb.2016.00076 PMC4740390 26870029

[22] Wu X, Holmfeldt K, Hubalek V, Lundin D, Åström M, Bertilsson S, Dopson M: Microbial metagenomes from three aquifers in the Fennoscandian Shield terrestrial deep biosphere reveal metabolic partitioning among populations. ISME J. 2015;10:1192–203. 10.1038/ismej.2015.185 PMC5029217 26484735

[23] Bagnoud A, Chourey K, Hettich RL, de Bruijn I, Andersson AF, Leupin OX, Schwyn B, Bernier-Latmani R (2016). Reconstructing a hydrogen-driven microbial metabolic network in Opalinus Clay rock. Nat Commun.

[24] Wanger G, Southam G, Onstott TC (2006). Structural and chemical characterization of a natural fracture surface from 2.8 kilometers below land surface: biofilms in the deep subsurface. Geomicrobiol J.

[25] Pfiffner SM, Cantu JM, Smithgall A, Peacock AD, White DC, Moser DP, Onstott TC, van Heerden E (2006). Deep subsurface microbial biomass and community structure in Witwatersrand Basin mines. Geomicrobiol J.

[26] Jägevall S, Rabe L, Pedersen K (2011). Abundance and diversity of biofilms in natural and artificial aquifers of the Äspö Hard Rock Laboratory, Sweden. Microb Ecol.

[27] Gimeno MJ, Auqué LF, Acero P, Gómez JB (2014). Hydrogeochemical characterisation and modelling of groundwaters in a potential geological repository for spent nuclear fuel in crystalline rocks (Laxemar, Sweden). Appl Geochem.

[28] Mathurin FA, Astrom ME, Laaksoharju M, Kalinowski BE, Tullborg EL (2012). Effect of tunnel excavation on source and mixing of groundwater in a coastal granitoidic fracture network. Environ Sci Technol.

[29] Mathurin FA, Drake H, Tullborg E-L, Berger T, Peltola P, Kalinowski BE, Åström ME (2014). High cesium concentrations in groundwater in the upper 1.2 km of fractured crystalline rock—Influence of groundwater origin and secondary minerals. Geochim Cosmochim Acta.

[30] Mathurin FA, Åström ME, Drake H, Maskenskaya OM, Kalinowski BE (2014). REE and Y in groundwater in the upper 1.2 km of Proterozoic granitoids (Eastern Sweden)—Assessing the role of composition and origin of groundwaters, geochemistry of fractures, and organic/inorganic aqueous complexation. Geochim Cosmochim Acta.

[31] Louvat D, Michelot JL, Aranyossy JF (1999). Origin and residence time of salinity in the Äspö groundwater system. Appl Geochem.

[32] Pedersen K, Bengtsson AF, Edlund JS, Eriksson LC (2014). Sulphate-controlled diversity of subterranean microbial communities over depth in deep groundwater with opposing gradients of sulphate and methane. Geomicrobiol J.

[33] Drake H, Tullborg E-L, Page L (2009). Distinguishing multiple events of fracture mineralisations related to far-field orogenic effects in Paleoproterozoic crystalline rocks, Simpevarp area, SE Sweden. Lithos.

[34] Drake H, Tullborg EL (2009). Detecting the near-surface redox front in crystalline bedrock using fracture mineral distribution, geochemistry and U-series disequilibrium. Appl Geochem.

[35] Andersen CR, James RE, Fru EC, Kennedy MA, Pedersen K (2006). In situ ecological development of a bacteriogenic iron oxide-producing microbial community from a subsurface granitic rock environment. Geobiology.

[36] Pedersen K, Ekendahl S (1992). Assimilation of CO_2_ and introduced organic compounds by bacterial communities in groundwater from southeastern Sweden deep crystalline bedrock. Microb Ecol.

[37] Hall-Stoodley L, Costerton JW, Stoodley P (2004). Bacterial biofilms: from the natural environment to infectious diseases. Nat Rev Microbiol.

[38] Edlund J, Rabe L, Bengtsson A, Hallbeck B, Eriksson L, Johansson J, Johansson L, Pedersen K. Understanding microbial reduction of sulphate to sulphide in deep Olkiluoto groundwater. Compilation and interpretation of three consecutive Sulphate Reduction Experiments (SURE) performed during 2010 - 2014. In *Posiva Working Report 2016-48*. pp. 1-164; 2016.

[39] Pedersen K. The MICROBE project. Achievements of a 10-year research program. SKB report R-13-49. Stockholm Sweden. pp. 1-40; 2013.

[40] Hubalek V, Wu X, Eiler A, Buck M, Heim C, Dopson M, Bertilsson S, Ionescu D (2016). Connectivity to the surface determines diversity patterns in subsurface aquifers of the Fennoscandian Shield. ISME J.

[41] Rocca JD, Hall EK, Lennon JT, Evans SE, Waldrop MP, Cotner JB, Nemergut DR, Graham EB, Wallenstein MD (2015). Relationships between protein-encoding gene abundance and corresponding process are commonly assumed yet rarely observed. ISME J.

[42] Ekendahl S, Pedersen K (1994). Carbon transformations by attached bacterial populations in granitic groundwater from deep crystalline bed-rock of the Stripa research mine. Microbiology.

[43] Holscher T, Bartels B, Lin YC, Gallegos-Monterrosa R, Price-Whelan A, Kolter R, Dietrich LE, Kovacs AT (2015). Motility, chemotaxis and aerotaxis contribute to competitiveness during bacterial pellicle biofilm development. J Mol Biol.

[44] Bogino PC, de las Mercedes Oliva M, Sorroche FG, Giordano W (2013). The role of bacterial biofilms and surface components in plant-cacterial associations. Int J Mo Sci.

[45] Flemming H-C, Wingender J (2010). The biofilm matrix. Nat Rev Microbiol.

[46] Perez-Rodriguez I, Bolognini M, Ricci J, Bini E, Vetriani C (2015). From deep-sea volcanoes to human pathogens: a conserved quorum-sensing signal in Epsilonproteobacteria. ISME J.

[47] Kojima H, Fukui M (2010). *Sulfuricella denitrificans* gen. nov., sp. nov., a sulfur-oxidizing autotroph isolated from a freshwater lake. Int J Syst Evol Bacteriol.

[48] Liu J, Wang Z, Belchik SM, Edwards MJ, Liu C, Kennedy DW, Merkley ED, Lipton MS, Butt JN, Richardson DJ, et al: Identification and characterization of MtoA: A decaheme *c*-type cytochrome of the neutrophilic Fe(II)-oxidizing bacterium *Sideroxydans lithotrophicus* ES-1. Front Microbiol. 2012;3. doi:10.3389/fmicb.2012.00037. 10.3389/fmicb.2012.00037 PMC3274759 22347878

[49] Pedersen K, Bengtsson A, Edlund J, Rabe L, Hazen T, Chakraborty R, Goodwin L, Shapiro N. Complete genome sequence of the subsurface, mesophilic sulfate-reducing bacterium *Desulfovibrio aespoeensis* Aspo-2. Genome Announc. 2014;2. doi:10.1128/genomeA.00509-00514. 10.1128/genomeA.00509-14 PMC4038888 24874683

[50] Beller HR, Chain PS, Letain TE, Chakicherla A, Larimer FW, Richardson PM, Coleman MA, Wood AP, Kelly DP (2006). The genome sequence of the obligately chemolithoautotrophic, facultatively anaerobic bacterium *Thiobacillus denitrificans*. J Bacteriol.

[51] Nielsen ME, Fisk MR, Istok JD, Pedersen K (2006). Microbial nitrate respiration of lactate at in situ conditions in ground water from a granitic aquifer situated 450 m underground. Geobiology.

[52] Ström A, Andersson J, Skagius K, Winberg A (2008). Site descriptive modelling during characterization for a geological repository for nuclear waste in Sweden. Appl Geochem.

[53] Pedersen K (1997). Microbial life in deep granitic rock. FEMS Microbiol Rev.

[54] Laaksoharju M, Gascoyne M, Gurban I (2008). Understanding groundwater chemistry using mixing models. Appl Geochem.

[55] Smellie JAT, Laaksoharju M, Wikberg P (1995). Äspö, SE Sweden—a natural groundwater-flow model derived from hydrogeological observations. J Hydrol.

[56] Alakangas LJ, Mathurin FA, Faarinen M, Wallin B, Åström ME (2014). Sampling and characterizing rare earth elements in groundwater in deep-lying fractures in granitoids under in situ high-pressure and low-redox conditions. Aquat Geochem.

[57] Marteinsson VT, Runarsson A, Stefansson A, Thorsteinsson T, Johannesson T, Magnusson SH, Reynisson E, Einarsson B, Wade N, Morrison HG, Gaidos E (2013). Microbial communities in the subglacial waters of the Vatnajokull ice cap, Iceland. ISME J.

[58] Huber JA, Mark Welch DB, Morrison HG, Huse SM, Neal PR, Butterfield DA, Sogin ML (2007). Microbial population structures in the deep marine biosphere. Science.

[59] Lundin S, Stranneheim H, Pettersson E, Klevebring D, Lundeberg J (2010). Increased throughput by parallelization of library preparation for massive sequencing. PLoS ONE.

[60] Borgström E, Lundin S, Lundeberg J (2011). Large scale library generation for high throughput sequencing. PLoS ONE.

[61] Huse SM, Huber JA, Morrison HG, Sogin ML, Welch DM (2007). Accuracy and quality of massively parallel DNA pyrosequencing. Genome Biol.

[62] Edgar RC, Haas BJ, Clemente JC, Quince C, Knight R (2011). UCHIME improves sensitivity and speed of chimera detection. Bioinformatics.

[63] Edgar RC (2010). Search and clustering orders of magnitude faster than BLAST. Bioinformatics.

[64] Joshi NA, Fass JN: Sickle. A sliding-window, adaptive, quality-based trimming tool for FastQ files. Version 1.33 edition. 2011. https://github.com/najoshi/sickle.

[65] Boisvert S, Raymond F, Godzaridis E, Laviolette F, Corbeil J (2012). Ray Meta: scalable de novo metagenome assembly and profiling. Genome Biol.

[66] Alneberg J, Bjarnason BS, de Bruijn I, Schirmer M, Quick J, Ijaz UZ, Lahti L, Loman NJ, Andersson AF, Quince C (2014). Binning metagenomic contigs by coverage and composition. Nat Methods.

[67] Darling AE, Jospin G, Lowe E, Matsen FA, Bik HM, Eisen JA (2014). PhyloSift: phylogenetic analysis of genomes and metagenomes. PeerJ.

[68] Seemann T (2014). Prokka: rapid prokaryotic genome annotation. Bioinformatics.

[69] Li D, Liu CM, Luo R, Sadakane K, Lam TW (2015). MEGAHIT: an ultra-fast single-node solution for large and complex metagenomics assembly via succinct de Bruijn graph. Bioinformatics.

[70] Quinlan AR, Hall IM (2010). BEDTools: a flexible suite of utilities for comparing genomic features. Bioinformatics.

[71] Eydal HS, Pedersen K (2007). Use of an ATP assay to determine viable microbial biomass in Fennoscandian Shield groundwater from depths of 3-1000 m. J Microbiol Methods.

