# Supplementary figures and images for: Potential for hydrogen-oxidizing chemolithoautotrophic and diazotrophic populations to initiate biofilm formation in oligotrophic, deep terrestrial subsurface waters
Source: Microbiome. 2017 Mar 23;5:37. doi: 10.1186/s40168-017-0253-y (PMC5364579; doi:10.1186/s40168-017-0253-y)

**Figure S1.** A flow cell connected to a borehole in the Äspö HRL tunnel.

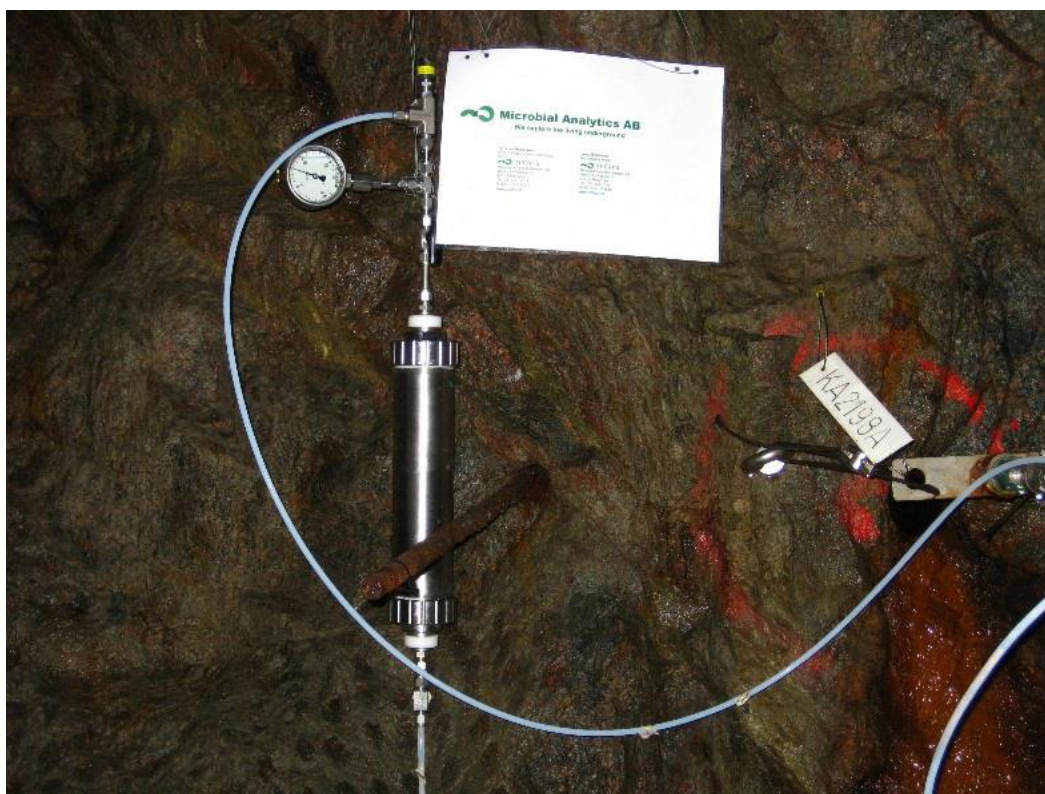

Supplement: Supplementary file 1 — A flow cell connected to a borehole in the Äspö HRL tunnel. (PDF 195 kb) [file 40168_2017_253_MOESM1_ESM.pdf]

● KF0069A01 (old saline)  
◆ KA2198A (modern marine)

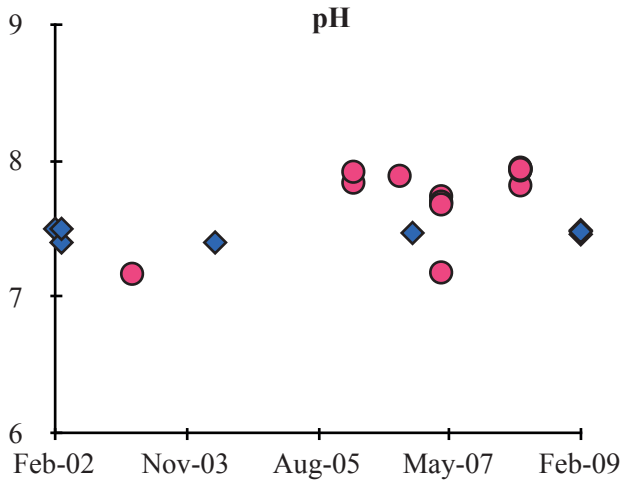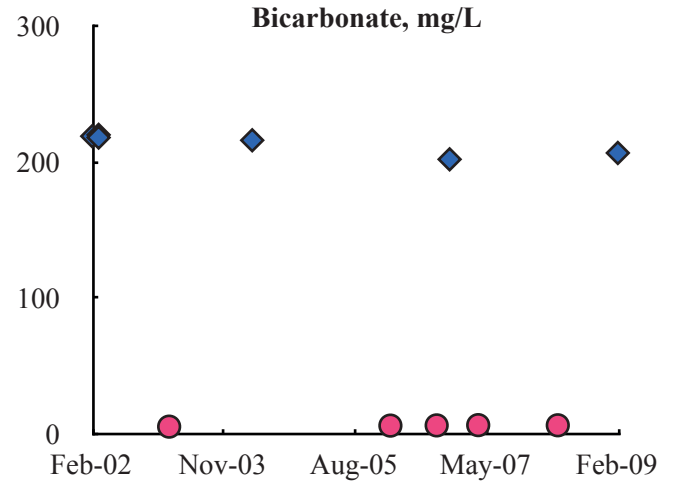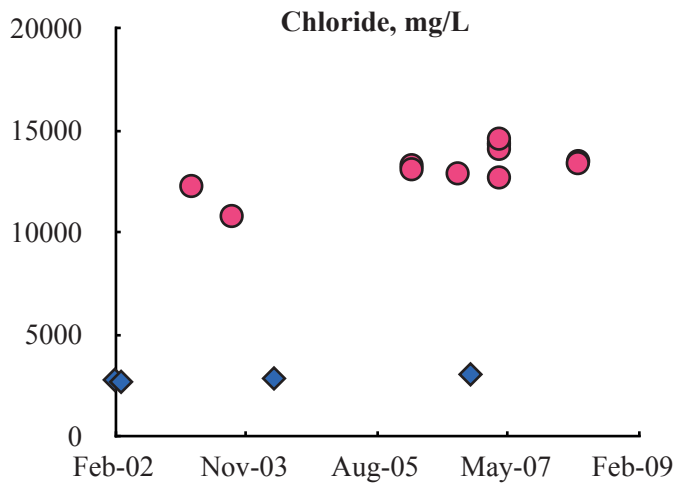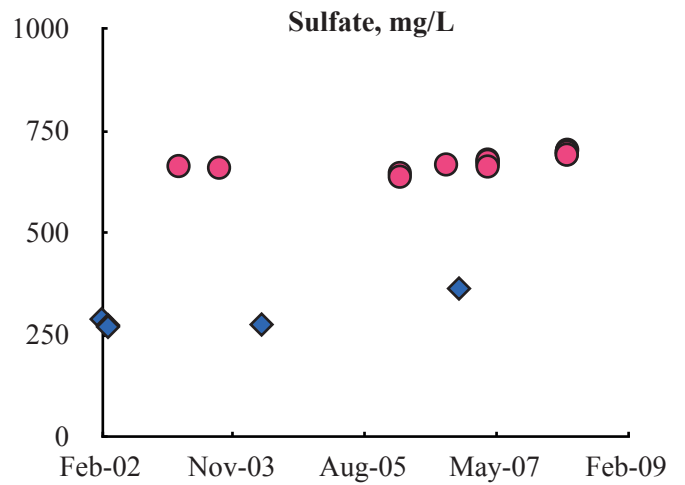

Supplement: Supplementary file 2 — Geochemical measurements over time showing the stability of the two groundwater systems. (PDF 877 kb) [file 40168_2017_253_MOESM2_ESM.pdf]

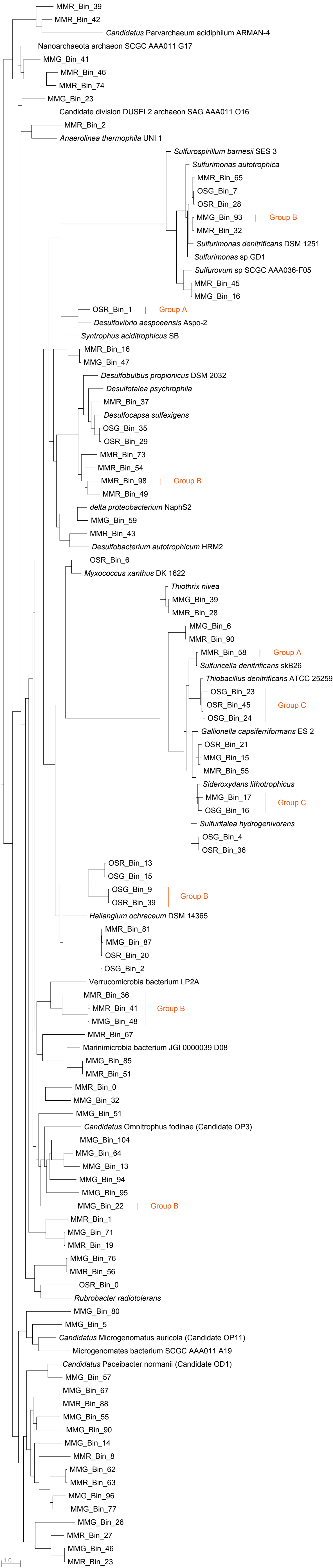

Supplement: Supplementary file 9 — Whole-genome phylogenetic tree of the relationship between the CONCOCT bins visualized by Archaeopteryx. Scale bar equals 1.0%. (PDF 180 kb) [file 40168_2017_253_MOESM9_ESM.pdf]

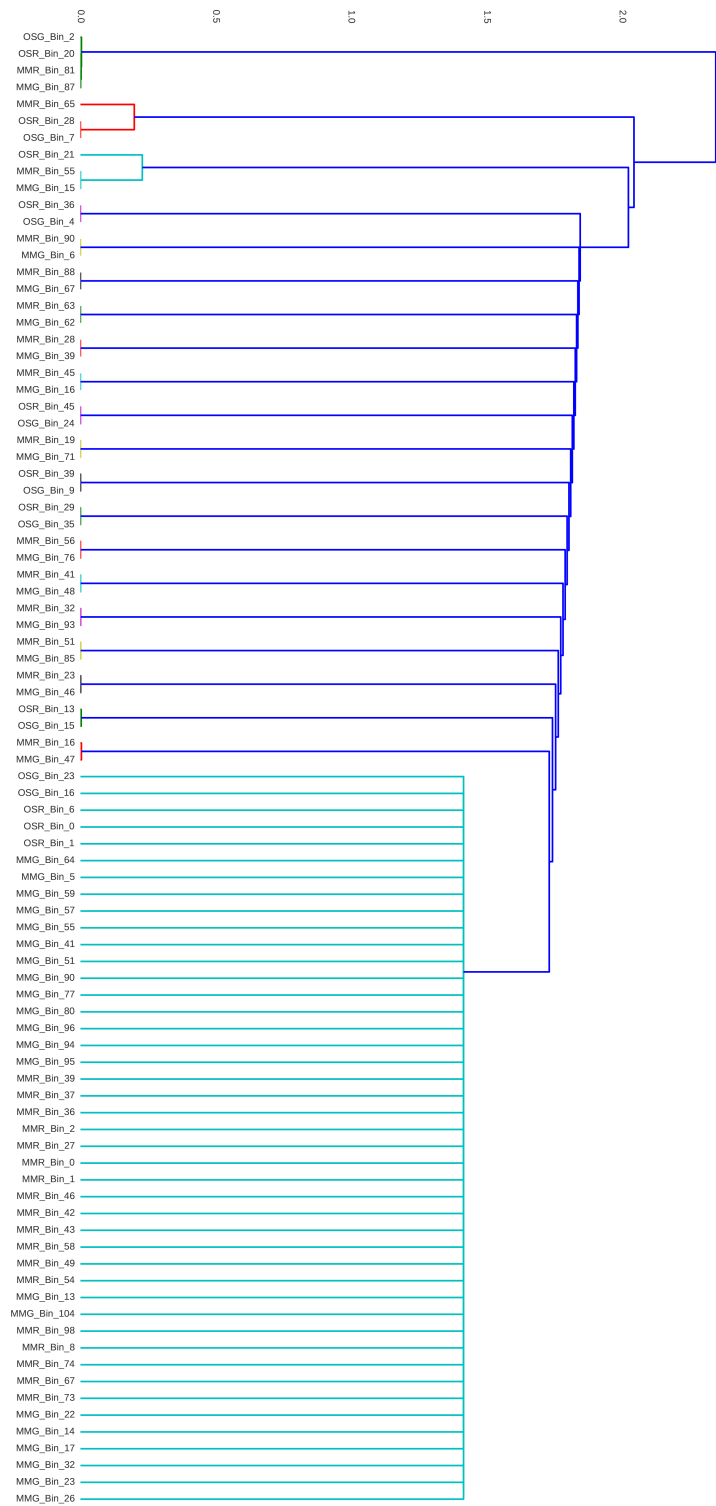

Supplement: Supplementary file 10 — Dendrogram of alignment from all near-complete reconstructed genomes (clustered bins showing >50% of the aligned base is the same). (PDF 578 kb) [file 40168_2017_253_MOESM10_ESM.pdf]

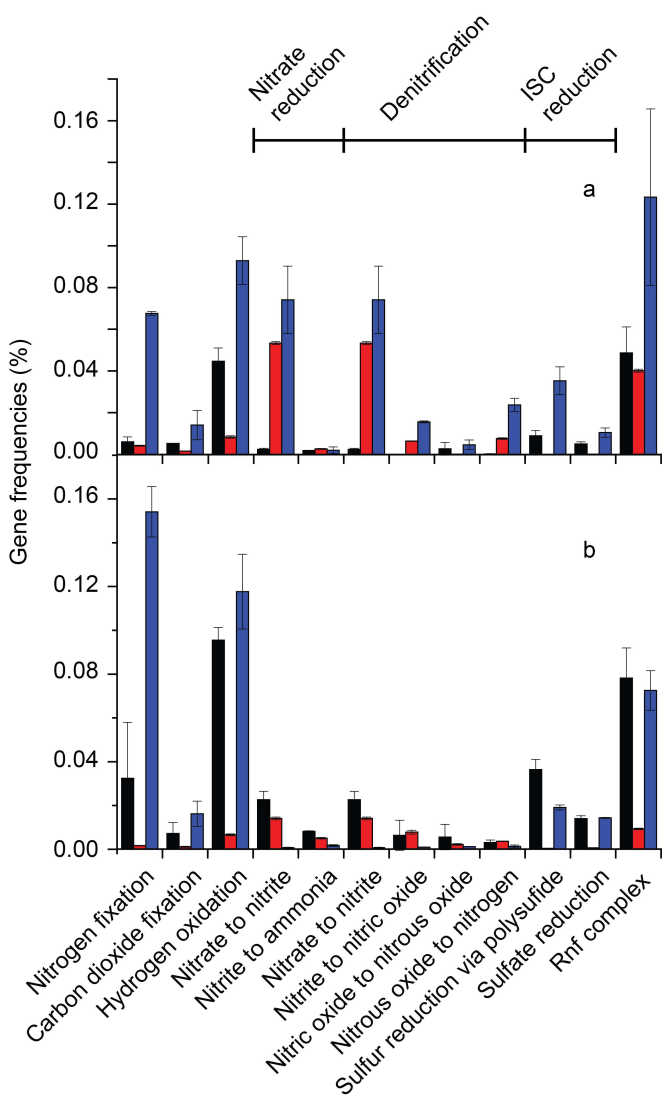

Supplement: Supplementary file 12 — Gene frequencies for selected characteristics (as defined in Table S6) in the modern marine (A) and old saline waters (B). Color coding: large (>0.22 μm) planktonic cells (black), small (<0.22 μm) planktonic cells (red), and biofilm cells (blue). Error bars denote standard deviations of duplicate samples. Abbreviation: ISC, inorganic sulfur compound. (PDF 405 kb) [file 40168_2017_253_MOESM12_ESM.pdf]
