# Supplementary material for: Potential for hydrogen-oxidizing chemolithoautotrophic and diazotrophic populations to initiate biofilm formation in oligotrophic, deep terrestrial subsurface waters
Source: Microbiome. 2017 Mar 23;5:37. doi: 10.1186/s40168-017-0253-y (PMC5364579; doi:10.1186/s40168-017-0253-y)
Supplement: Supplementary file 3 — Samples from groundwater with the corresponding flow cell biofilm samples. The table shows decreasing diversity by depth below sea level (mbsl). Amounts of extracted double-stranded bacterial DNA analyzed fluorometrically using the Stratagene MX3005p fluorometer with MXPro software and the Quant-it Picogreen reagent kit from Molecular Probes. (PDF 13 kb) [file 40168_2017_253_MOESM3_ESM.pdf]

Table S1. Samples from groundwater with the corresponding flow cell biofilm samples. The table shows decreasing diversity by depth below sea level (mbsl). Amounts of extracted double-stranded bacterial DNA analyzed fluorometrically using the Stratagene MX3005p fluorometer with MXPro software and the Quant-it Picogreen reagent kit from Molecular Probes.

Sample	Depth (mbsl[a])	Sample Type	Growth (days)	Total water volume (L)[b]	dsDNA (ng)	Number sequences[c]
KA2198A_1	300	Groundwater	-	192	268	15380
KA2198A_garnet	300	Biofilm	33	13900	17	16388
KA2198A_glass	300	Biofilm	33	13900	101	22823
KF0069A01_2	450	Groundwater	-	198	12.8	13241
KF0069A01_garnet	450	Biofilm	33	77700	6.9	13551
KF0069A01_glass	450	Biofilm	33	77700	4.7	14630

[a] mbsl, meters below sea level

[b] total borehole water passed through the flow cells during the 33 days

[c] number of 16S rRNA gene sequences obtained from the Illumina MiSeq sequencing
