# Supplementary material for: Potential for hydrogen-oxidizing chemolithoautotrophic and diazotrophic populations to initiate biofilm formation in oligotrophic, deep terrestrial subsurface waters
Source: Microbiome. 2017 Mar 23;5:37. doi: 10.1186/s40168-017-0253-y (PMC5364579; doi:10.1186/s40168-017-0253-y)
Supplement: Supplementary file 4 — Sequencing information for the four metagenomes. (PDF 60 kb) [file 40168_2017_253_MOESM4_ESM.pdf]

Table S2. Sequencing information for the four metagenomes.

Sample ID	Borehole name	Water type	Number raw reads[a]	Number Trimmed reads[a]	Number contigs (>=1000bp)	% Of reads binned into a near complete genome	Number of bins
MMR	KA2198A_garnet	Modern marine	157472854	154061601	48857	49.68	33
MMG	KA2198A_glass	Modern marine	144739126	141551476	44801	37.13	35
OSR	KF0069A01_garnet	Old saline	141472772	138470966	10496	45.98	11
OSG	KF0069A01_glass	Old saline	170306638	165931084	5671	44.81	9

[a] Values for MMR, MMG, OSR & OSG are totals for the duplicate sequencing.
