# Supplementary material for: Potential for hydrogen-oxidizing chemolithoautotrophic and diazotrophic populations to initiate biofilm formation in oligotrophic, deep terrestrial subsurface waters
Source: Microbiome. 2017 Mar 23;5:37. doi: 10.1186/s40168-017-0253-y (PMC5364579; doi:10.1186/s40168-017-0253-y)
Supplement: Supplementary file 5 — Sequencing information for each approved phylogenetic bin from the metagenomes. (PDF 95 kb) [file 40168_2017_253_MOESM5_ESM.pdf]

Table S3. Sequencing information for each approved phylogenetic bin from the metagenomes.

Bins	Estimated genome size (bp)	No. contigs	n50[a]	GC%	No. single copy genes	No. duplicated single copy genes	Contig length cut-off	Mapped reads (%)[b]	Related phylogeny
**Modern marine water biofilm on rock**									
MMR_Bin_0	3542867	530	142	41.1	35/36	1/36	2000	0.20	Unclassified bacteria
MMR_Bin_16	2876130	465	119	56.5	34/36	0/36	2000	0.13	Deltaproteobacteria
MMR_Bin_19	1841450	169	81	42.4	34/36	0/36	2000	0.28	Unclassified bacteria
MMR_Bin_1	1752131	180	57	50.9	34/36	1/36	2000	0.08	Unclassified bacteria
MMR_Bin_23	1571892	152	51	46.5	34/36	0/36	2000	2.53	Candidatus OD1
MMR_Bin_27	1812957	375	95	40.4	31/36	1/36	2000	0.05	Candidatus OD1
MMR_Bin_28	4144501	393	166	54.4	36/36	1/36	2000	0.28	Gammaproteobacteria
MMR_Bin_2	6658576	793	259	63.8	36/36	0/36	2000	0.51	Chloroflexi/Dehalococcoidia
MMR_Bin_32	2679511	287	98	36.0	36/36	0/36	2000	10.01	Epsilonproteobacteria
MMR_Bin_36	3507257	472	126	63.8	32/36	1/36	2000	0.15	Verrucomicrobia
MMR_Bin_37	3402314	331	153	49.8	36/36	0/36	2000	0.55	Deltaproteobacteria
MMR_Bin_39	1246058	122	40	37.5	32/36	1/36	2000	0.04	Unclassified archaea
MMR_Bin_41	4085162	381	130	54.9	34/36	0/36	2000	0.22	Verrucomicrobia
MMR_Bin_42	1416895	135	46	39.2	34/36	0/36	2000	0.06	Unclassified archaea
MMR_Bin_43	3077456	404	114	44.2	34/36	0/36	2000	0.14	Deltaproteobacteria
MMR_Bin_45	2868529	257	98	35.3	36/36	0/36	2000	2.81	Epsilonproteobacteria
MMR_Bin_46	1298135	134	43	35.5	34/36	0/36	2000	0.16	Unclassified archaea
MMR_Bin_49	4217441	403	178	50.8	36/36	0/36	2000	1.28	Deltaproteobacteria
MMR_Bin_51	4024447	441	143	42.2	36/36	0/36	2000	0.49	Fibrobacteres/Acidobacteria
MMR_Bin_54	3869690	368	165	48.7	36/36	1/36	2000	0.46	Deltaproteobacteria
MMR_Bin_55	2258877	220	108	56.7	36/36	0/36	2000	1.94	Betaproteobacteria
MMR_Bin_56	3888959	363	164	64.3	36/36	0/36	2000	0.35	Actinobacteria
MMR_Bin_58	3477281	432	116	56.0	32/36	0/36	2000	0.13	Betaproteobacteria
MMR_Bin_63	1381669	117	53	35.7	32/36	0/36	2000	0.19	Candidatus OD1

MMR_Bin_65	4207466	699	185	36.6	34/36	2/36	2000	16.95	Epsilonproteobacteria
MMR_Bin_67	4998168	846	226	55.0	34/36	0/36	2000	0.19	Unclassified bacteria
MMR_Bin_73	3701674	329	132	47.6	35/36	0/36	2000	0.28	Deltaproteobacteria
MMR_Bin_74	2219889	237	73	31.7	32/36	1/36	2000	0.8	Unclassified Archaea
MMR_Bin_81	6140676	686	213	64.4	35/36	0/36	2000	0.38	Deltaproteobacteria
MMR_Bin_88	1513438	121	50	40.7	32/36	1/36	2000	0.21	Candidatus OD1
MMR_Bin_8	883055	146	42	50.4	32/36	0/36	2000	0.03	Candidatus OD1
MMR_Bin_90	4174888	386	154	60.4	36/36	0/36	2000	6.99	Gammaproteobacteria
MMR_Bin_98	3889192	366	167	52.7	36/36	0/36	2000	0.81	Deltaproteobacteria
**Modern marine water biofilm on glass**									
MMG_Bin_104	2054129	355	101	43.2	31/36	0/36	2000	0.07	Candidatus OP3
MMG_Bin_13	2098779	194	69	39.2	34/36	0/36	2000	0.14	Candidatus OP3
MMG_Bin_14	622360	141	46	29.8	31/36	0/36	2000	0.04	Candidatus OD1
MMG_Bin_15	2267813	216	105	56.6	35/36	0/36	2000	2.59	Betaproteobacteria
MMG_Bin_16	2852884	259	105	35.4	36/36	0/36	2000	3.99	Epsilonproteobacteria
MMG_Bin_17	3252966	317	155	59.2	36/36	0/36	2000	3.10	Betaproteobacteria
MMG_Bin_22	1555980	157	70	37.3	36/36	0/36	2000	1.06	Unclassified bacteria
MMG_Bin_23	1726469	343	100	38.1	34/36	0/36	2000	0.06	Unclassified archaea
MMG_Bin_26	1475530	136	57	42.5	34/36	2/36	2000	0.11	Candidatus OD1
MMG_Bin_32	4344203	669	174	43.3	34/36	2/36	2000	1.66	Unclassified bacteria
MMG_Bin_39	4193981	384	164	54.4	35/36	0/36	2000	1.99	Gammaproteobacteria
MMG_Bin_41	1557818	149	54	36.0	36/36	0/36	2000	0.12	Unclassified archaea
MMG_Bin_46	1528930	150	54	46.3	34/36	0/36	2000	0.93	Candidatus OD1
MMG_Bin_47	3568108	644	158	55.8	35/36	2/36	2000	0.4	Deltaproteobacteria
MMG_Bin_48	4030226	357	145	55.0	34/36	0/36	2000	0.57	Verrucomicrobia
MMG_Bin_51	2534356	235	90	37.3	34/36	0/36	2000	0.18	Unclassified bacteria
MMG_Bin_55	1590175	136	51	38.2	33/36	1/36	2000	0.25	Candidatus OD1
MMG_Bin_57	610983	58	18	33.6	31/36	0/36	2000	0.03	Candidatus OD1
MMG_Bin_59	1795855	481	173	50.6	31/36	0/36	2000	0.07	Deltaproteobacteria

MMG_Bin_5	1093618	95	36	40.2	33/36	0/36	2000	0.06	Candidatus OP11
MMG_Bin_62	1633848	229	59	35.9	34/36	0/36	2000	0.08	Candidatus OD1
MMG_Bin_64	1601178	268	71	41.5	31/36	1/36	2000	0.06	Candidatus OP3
MMG_Bin_67	1499820	116	46	40.8	31/36	0/36	2000	0.11	Candidatus OD1
MMG_Bin_6	4164270	390	158	60.4	36/36	0/36	2000	6.10	Gammaproteobacteria
MMG_Bin_71	1910255	180	85	42.4	34/36	1/36	2000	0.13	Unclassified bacteria
MMG_Bin_76	3831845	403	131	64.3	36/36	0/36	2000	0.20	Actinobacteria
MMG_Bin_77	948620	121	34	36.8	34/36	1/36	2000	0.05	Candidatus OD1
MMG_Bin_80	755777	69	35	37.9	33/36	0/36	2000	0.05	Candidatus OP11
MMG_Bin_85	3863255	371	140	42.4	36/36	0/36	2000	0.27	Fibrobacteres/Acidobacteria
MMG_Bin_87	5530026	938	262	65.1	34/36	0/36	2000	0.26	Deltaproteobacteria
MMG_Bin_90	1415040	135	46	40.1	34/36	0/36	2000	0.18	Candidatus OD1
MMG_Bin_93	2833704	395	104	35.8	34/36	0/36	2000	11.73	Epsilonproteobacteria
MMG_Bin_94	1902438	188	64	41.7	34/36	0/36	2000	0.10	Candidatus OP3
MMG_Bin_95	2421288	286	83	45.1	33/36	0/36	2000	0.32	Candidatus OP3
MMG_Bin_96	1211867	111	37	39.6	32/36	0/36	2000	0.07	Candidatus OD1
**Old saline water biofilm on rock**									
OSR_Bin_0	2069139	635	186	68.5	31/36	0/36	1000	0.08	Actinobacteria
OSR_Bin_13	5807397	773	198	62.9	33/36	0/36	1000	1.22	Deltaproteobacteria
OSR_Bin_1	3141997	296	121	66.9	36/36	0/36	1000	0.22	Deltaproteobacteria
OSR_Bin_20	6104586	1045	243	65.2	36/36	0/36	1000	0.47	Deltaproteobacteria
OSR_Bin_21	2180029	369	90	58.0	33/36	0/36	1000	0.09	Betaproteobacteria
OSR_Bin_28	2508133	237	96	37.2	36/36	0/36	1000	32.90	Epsilonproteobacteria
OSR_Bin_29	4513125	470	177	48.1	36/36	0/36	1000	2.03	Deltaproteobacteria
OSR_Bin_36	3046543	300	147	63.1	36/36	0/36	1000	7.15	Betaproteobacteria
OSR_Bin_39	5615785	522	223	42.0	36/36	1/36	1000	0.65	Deltaproteobacteria
OSR_Bin_45	3762603	334	159	63.8	33/36	0/36	1000	1.71	Betaproteobacteria
OSR_Bin_6	9662296	1232	345	68.9	33/36	2/36	1000	0.46	Deltaproteobacteria

**Old saline water biofilm on glass**									
OSG_Bin_15	5535056	736	192	63.7	32/36	0/36	1000	0.34	Deltaproteobacteria
OSG_Bin_16	3268064	309	138	57.5	36/36	0/36	1000	0.29	Betaproteobacteria
OSG_Bin_23	2907113	639	159	63.4	31/36	0/36	1000	0.38	Betaproteobacteria
OSG_Bin_24	3471611	329	147	63.8	36/36	2/36	1000	2.15	Betaproteobacteria
OSG_Bin_2	6193975	1063	231	65.7	35/36	0/36	1000	1.14	Deltaproteobacteria
OSG_Bin_35	4946118	461	213	48.1	35/36	0/36	1000	2.27	Deltaproteobacteria
OSG_Bin_4	3050447	297	143	63.1	36/36	0/36	1000	13.1	Betaproteobacteria
OSG_Bin_7	2435414	234	104	37.2	36/36	0/36	1000	24.80	Epsilonproteobacteria
OSG_Bin_9	5430639	688	203	42.0	36/36	1/36	1000	0.34	Deltaproteobacteria

[a] n50, number of the largest contigs that sum up to 50% of the total sum of bases

[b]Mapped reads (%) is the average percentage for duplicate sequencing from respective metagenomes
