# Supplementary material for: Potential for hydrogen-oxidizing chemolithoautotrophic and diazotrophic populations to initiate biofilm formation in oligotrophic, deep terrestrial subsurface waters
Source: Microbiome. 2017 Mar 23;5:37. doi: 10.1186/s40168-017-0253-y (PMC5364579; doi:10.1186/s40168-017-0253-y)
Supplement: Supplementary file 6 — Extracted ATP and estimated cell numbers from the planktonic cells in the water phase as well as from the biofilms formed on garnet grains and glass beads. Values from this study presented as averages of three replicates ± SD. (PDF 78 kb) [file 40168_2017_253_MOESM6_ESM.pdf]

Table S4. Extracted ATP and estimated cell numbers from the planktonic cells in the water phase as well as from the biofilms formed on garnet grains and glass beads. Values from this study presented as averages of three replicates ± SD.

	Borehole	
	KA2198A (MM)	KF0069A01 (OS)
Planktonic ATP (amol/cm^3)	ND[a]	ND
Planktonic estimated (cells/cm^3)	2.76×10^{4} [b]	ND
Garnet ATP (amol/cm^2)	$1.97 \times 10^6 \pm 5.31 \times 10^4$	$7.95 \times 10^3 \pm 72$
Garnet estimated (cells/cm^2)	$4.58 \times 10^6 \pm 1.24 \times 10^5$	$1.9 \times 10^4 \pm 173$
Glass ATP (amol/cm^2)	$8.47 \times 10^7 \pm 2.54 \times 10^4$	$1.86 \times 10^3 \pm 139$
Glass estimated (cells/cm^2)	$1.97 \times 10^8 \pm 5.91 \times 10^4$	$4.32 \times 10^3 \pm 324$

[a] ND, no data available

[b] Data from 2003 and published in: Andersen et al. (2006). *In situ* ecological development of a bacteriogenic iron oxide-producing microbial community from a subsurface granitic rock environment. *Geobiology* **4:** 29-42.
