# Supplementary material for: Potential for hydrogen-oxidizing chemolithoautotrophic and diazotrophic populations to initiate biofilm formation in oligotrophic, deep terrestrial subsurface waters
Source: Microbiome. 2017 Mar 23;5:37. doi: 10.1186/s40168-017-0253-y (PMC5364579; doi:10.1186/s40168-017-0253-y)

Figure S3. Rarefaction curves for bacterial 16S rRNA gene v4v6 dataset. Each curve represents a single sample and sampling occasion.

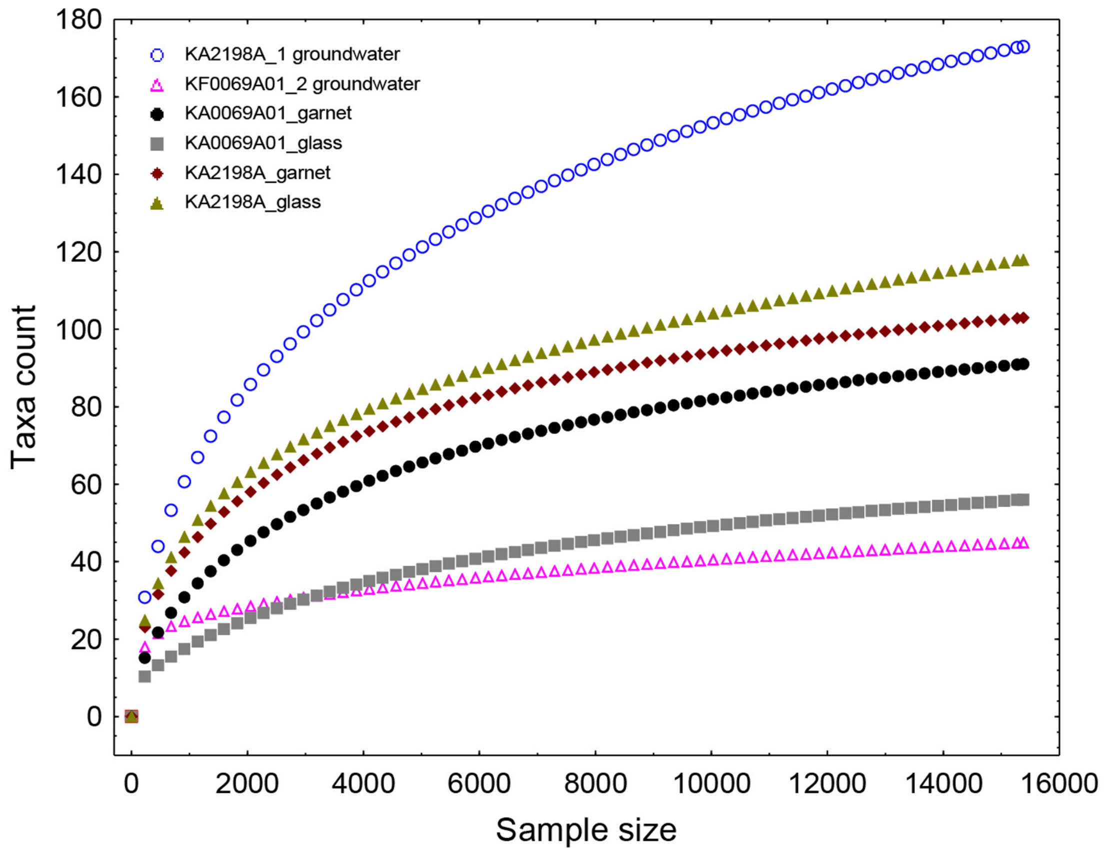

Supplement: Supplementary file 7 — Rarefaction curves for bacterial 16S rRNA gene v4v6 dataset. Each curve represents a single sample and sampling occasion. (PDF 422 kb) [file 40168_2017_253_MOESM7_ESM.pdf]
