# Supplementary material for: Potential for hydrogen-oxidizing chemolithoautotrophic and diazotrophic populations to initiate biofilm formation in oligotrophic, deep terrestrial subsurface waters
Source: Microbiome. 2017 Mar 23;5:37. doi: 10.1186/s40168-017-0253-y (PMC5364579; doi:10.1186/s40168-017-0253-y)
Supplement: Supplementary file 8 — Species richness estimates (Chao1 and ACE) and diversity indices (Shannon-Weaver and Inverse Simpson) for the 16S rRNA gene sequencing. The >0.1 and >1% abundance taxa number was generated at genus level or the highest annotated rank. (PDF 14 kb) [file 40168_2017_253_MOESM8_ESM.pdf]

Table S5. Species richness estimates (Chao1 and ACE) and diversity indices (Shannon-Weaver and Inverse Simpson) for the 16S rRNA gene sequencing. The > 0.1 % and > 1 % abundance taxa number was generated at genus level or the highest annotated rank.

Sample	Sample type	Number taxa > 0% abundance	Number taxa > 0.1% abundance	Number taxa > 1% abundance	ACE	Chao	Shannon-Weaver	Inverse Simpson
KA2198A_1	Groundwater	229	55	13	264.4	373.86	2.14	3.52
KA2198A_glass	Biofilm	174	45	12	201.28	264.25	1.9	3.23
KA2198A_garnet	Biofilm	131	43	8	142.28	185.45	1.85	3.23
KF0069A01_2	Groundwater	53	22	13	62.53	95.67	1.8	3.38
KF0069A01_glass	Biofilm	89	14	7	105.5	125.3	1.39	2.89
KF0069A01_garnet	Biofilm	114	29	6	128.51	198.5	1.51	2.92
