# Supplementary material for: Potential for hydrogen-oxidizing chemolithoautotrophic and diazotrophic populations to initiate biofilm formation in oligotrophic, deep terrestrial subsurface waters
Source: Microbiome. 2017 Mar 23;5:37. doi: 10.1186/s40168-017-0253-y (PMC5364579; doi:10.1186/s40168-017-0253-y)
Supplement: Supplementary file 11 — Gene frequencies for selected characteristics in the modern marine (MM) and old saline waters (OS). Abbreviations: PL, planktonic large cells; PS, planktonic small cells; B, biofilm. (PDF 77 kb) [file 40168_2017_253_MOESM11_ESM.pdf]

		MMPL Average	MMPL STDEV	MMPS Average	MMPS STDEV	MMB Average	MMB STDEV		OSPL Average	OSPL STDEV	OSPS Average	OSPS STDEV	OSB Average	OSB STDEV
Nutrient assimilation	Nitrogen fixation (EC1.18.6.1/*nifKDH*)	0.0058366	0.0022361	0.0040849	0.0002176	0.0644743	0.0008270		0.0339325	0.0268290	0.0016518	0.0001065	0.1617069	0.0120858
	CO_2 fixation (EC 4.1.1.39 / *cbbLMS*)	0.0051139	0.0000001	0.0016098	0.0000574	0.0134957	0.0067003		0.0074770	0.0051327	0.0010616	0.0002579	0.0169254	0.0060619
Lithotrophy	H_2 oxidation (EC 1.12.1.3)	0.0426355	0.0060481	0.0080431	0.0005585	0.0885628	0.0109135		0.1001421	0.0060987	0.0068423	0.0004275	0.1234347	0.0179218
Nitrate reduction	Nitrate to nitrite	0.0023706	0.0005389	0.0509219	0.0006359	0.0706943	0.0153847		0.0236703	0.0039617	0.0146900	0.0006399	0.0006443	0.0002101
	Nitrite to ammonia	0.0018486	0.0001143	0.0026152	0.0002132	0.0019974	0.0014995		0.0084533	0.0002931	0.0051915	0.0003659	0.0016931	0.0003997
Denitrification	Nitrate to nitrite	0.0023706	0.0005389	0.0509219	0.0006359	0.0706943	0.0153847		0.0236703	0.0039617	0.0146900	0.0006399	0.0006443	0.0002101
	Nitrite to nitric oxide	0.0000112	0.0000159	0.0061762	0.0001041	0.0149376	0.0004587		0.0065806	0.0072161	0.0081018	0.0009254	0.0008909	0.0000678
	Nitric oxide to nitrous oxide	0.0026422	0.0029053	0.0002178	0.0000140	0.0044887	0.0021947		0.0057553	0.0061265	0.0022101	0.0003707	0.0011518	0.0000610
	Nitrous oxide to nitrogen	0.0003024	0.0001493	0.0073253	0.0003746	0.0226788	0.0030539		0.0031328	0.0011641	0.0037141	0.0001213	0.0012790	0.0006869
ISC reduction	Sulfate reduction (*dsv*)	0.0085997	0.0023589	0.0000435	0.0000023	0.0337212	0.0063663		0.0381083	0.0048927	0.0003367	0.0000672	0.0199269	0.0011809
	Sulfur reduction via polysufide (*psrA*)	0.0048623	0.0009666	0.0002006	0.0000110	0.0100413	0.0021088		0.0146013	0.0013374	0.0005935	0.0000214	0.0149493	0.0002605
Rnf	Rnf complex (*rnfABCDEG*)	0.0464476	0.0118796	0.0383452	0.0006602	0.1175997	0.0402553		0.0819939	0.0144482	0.0096715	0.0003599	0.0760368	0.0095131
Initial attachment	MCP	0.0350614	0.0092731	0.1096104	0.0017833	0.0532070	0.0151901		0.0865414	0.0047272	0.1258511	0.0047122	0.0623544	0.0067970
	Chemotaxis	0.0831862	0.0231639	0.1246461	0.0014119	0.1445441	0.0444235		0.1779158	0.0045676	0.1644306	0.0074631	0.1183404	0.0071274
	Flagellar	0.1134102	0.0350200	0.4095647	0.0099960	0.2782661	0.0795632		0.2279528	0.0551749	0.3864468	0.0106385	0.5524997	0.0942195
	Motor	0.0188276	0.0070808	0.0461101	0.0013894	0.0161732	0.0004077		0.0301430	0.0030199	0.0293664	0.0010314	0.0310861	0.0043633
EPS	Glucose	0.0782823	0.0010333	0.0686595	0.0027996	0.0630428	0.0079245		0.0619994	0.0074412	0.0738437	0.0017642	0.0641004	0.0017964
	Fructose	0.0486731	0.0039906	0.0588989	0.0009066	0.0864522	0.0057647		0.0921719	0.0058807	0.0520734	0.0016234	0.0727516	0.0042364
Secretion	Type I secretion	0.0127595	0.0030420	0.0403001	0.0005853	0.0181660	0.0007324		0.0288940	0.0030990	0.0084718	0.0001819	0.0168824	0.0009336
	Type II secretion	0.1069279	0.0084278	0.0488360	0.0010806	0.1975256	0.0117231		0.1545696	0.0101374	0.0753842	0.0006824	0.1600413	0.0021877
