# Supplementary material for: Potential for hydrogen-oxidizing chemolithoautotrophic and diazotrophic populations to initiate biofilm formation in oligotrophic, deep terrestrial subsurface waters
Source: Microbiome. 2017 Mar 23;5:37. doi: 10.1186/s40168-017-0253-y (PMC5364579; doi:10.1186/s40168-017-0253-y)
Supplement: Supplementary file 13 — Metabolic characteristics identified in the metagenomic bins from the two water types. The listed pathways are based upon BioCyc (http://biocyc.org/) and KEGG (http://www.genome.jp/kegg/). Additional pathways that were searched for but are not listed as they were negative in all cases include ferric reduction as a terminal electron acceptor; methanogenesis; aerobic and anaerobic ammonia oxidation; the reductive TCA cycle, incomplete TCA cycle, 3-hydroxypropanoate cycle, and reductive acetyl CoA pathway for CO2 fixation; and lipopolysaccharide production and export, type I and IV pili, autolysin gene atlE for release of extracellular DNA; and quorum sensing by acyl homoserine lactones and peptides. (PDF 100 kb) [file 40168_2017_253_MOESM13_ESM.pdf]

Table S7. Metabolic characteristics identified in the metagenomic bins from the two water types. The listed pathways are based upon BioCyc (http://biocyc.org/) and KEGG (http://www.genome.jp/kegg/). Additional pathways that were searched for but are not listed as they were negative in all cases include: ferric reduction as a terminal electron acceptor; methanogenesis; aerobic and anaerobic ammonia oxidation; the reductive TCA cycle, incomplete TCA cycle, 3-hydroxypropanoate cycle, and reductive acetyl CoA pathway for CO_2 fixation; and lipopolysaccharide production and export, type I and IV pili, autolysin gene *atlE* for release of extracellular DNA, and quorum sensing by acyl homoserine lactones and peptides.

		Metabolic pathways					Respiration & electron transport					Nutrient fixation		Biofilm formation			
Group	Bins	Pyruvate[a]	H_2 production[b]	Ethanol (I - IV)[c]	Formate to CO_2[d]	H_2[e]	Sulfate reduction[f]	Sulfur reduction[g]	Nitrate reduction[h]	Denitri-fication[i]	Rnf[j]	CBB cycle[k]	N_2 fixation	MCP & chemotaxis[l]	Flagella & motor[m]	EPS[n]	EPS secretion[o]
**Modern Marine Rock**																	
A	MMR_Bin_58	-	II/IV	-	-	III	+	-	Nitrite (pr)	-	+	+	-	+	+	F	II
B	MMR_Bin_36	-	II	-	-	-	-	-	-	-	+	-	-	-	-	F	II
	MMR_Bin_41	Acetate	II/IV	-	-	III	-	-	-	-	+	-	-	+	-	F	II
	MMR_Bin_98	-	VII	-	Pu	-	+	Pr	Nitrite (pr)	-	-	-	+	+	-	F	I
I	MMR_Bin_28	Acetate & lactate	II/III/VII	-	Pu	-	+	Pr	Nitrite	N_2 (pr)	+	+	+	-	-	G	-
	MMR_Bin_55	-	II/VII	-	-	-	+	-	-	-	+	+	+		-	F	-
	MMR_Bin_90	-	II	-	Pr	-	+	Pr	Nitrite (pr)	-	+	+	-	-	-	G	-
II	MMR_Bin_43	Propionate 6/7	II/III/IV	-	Pr	III	+	Pr	-	-	+	-	-	-	-	-	I
	MMR_Bin_67	Propionate 5/7	II/III	-	Pr	-	+	Pr	-	-	+	-	-	-	-	-	II
III	MMR_Bin_73	-	II/VII	-	-	-	+	Pr	-	-	-	-	+	+	-	-	-
	MMR_Bin_0	-	II/IV	-	Pr	III	+	-	-	-	-	-	-	-	-	F	-
	MMR_Bin_27	Lactate	III	-	-	-	+	-	-	-	-	-	-	-	-	-	-
IV	MMR_Bin_49	-	II	-	Pu	-	+	Pr	Nitrite (pr)	-	-	-	-	+	-	F	-
	MMR_Bin_37	Ethanol 2/3	II/VII	-	Pu	-	+	Pr	Ammonia (pr)	-	-	-	+	+	-	F	-
	MMR_Bin_54	-	II/VII	-	-	-	+	-	Nitrite	-	-	-	+	+	-	F	-
V	MMR_Bin_1	Acetate	IV	-	-	III	-	-	Nitrite	-	+	-	-	-	-	F	-
	MMR_Bin_32	Acetate & ethanol 2/3	VII	-	-	-	-	Pr	Nitrite (pr)	N_2 (pr)	-	-	+	+	-	-	II
	MMR_Bin_45	Acetate	III/IV/VII	-	-	III	-	-	Nitrite (pr)	-	-	-	+	-	-	F	-

	MMR_Bin_65	Acetate	II/IV/VII	-	-	III	-	Pr	Nitrite (pr)	-	-	-	+	-	-	F	-
VI	MMR_Bin_19	Acetate & lactate	II	-	-	-	-	-	-	-	+	-	-	-	-	-	-
	MMR_Bin_51	-	II/IV	-	-	III	-	-	-	-	+	-	-	-	-	-	-
	MMR_Bin_56	Lactate & ethanol 2/3	II/IV	II/III	Pr	III	-	-	-	-	+	-	-	+	-	F	-
	MMR_Bin_81	Propionate 5/7	II/IV	-	-	III	-	Pr	-	-	+	-	-	+	-	F	-
VII	MMR_Bin_42	-	-	-	-	-	-	-	-	-		+	-	-	-	-	-
Un-grouped	MMR_Bin_23	Lactate	III	-	-	-	-	-	-	-	-	-	-	-	-	-	-
	MMR_Bin_2	Lactate & ethanol 2/3	II/IV	-	Pr	III	-	-	-	-	-	-	-	+	-	-	-
	MMR_Bin_16	Propionate 5/7 & ethanol 2/3	IV	-	Pr	III	-	Pr	-	-	-	-	-	-	-	F	-
	MMR_Bin_46	Lactate	-	-	-	-	-	-	-	-	-	-	-	-	-	-	-
	MMR_Bin_63	Lactate	-	-	-	-	-	-	-	-	-	-	-	-	-	-	-
	MMR_Bin_74	Lactate	-	-	-	-	-	-	-	-	-	-	-	-	-	-	-
	MMR_Bin_88	-	II	-	-	-	-	-	-	-	-	-	-	-	-	-	-
	MMR_Bin_8	-	III/IV	-	-	III	-	-	-	-	-	-	-	-	-	-	-
	MMR_Bin_39	-	-	-	-	-	-	-	-	-	-	-	-	-	-	-	-
**Modern Marine Glass**																	
B	MMG_Bin_22	Lactate	-	-	-	-	-	-	-	-	+	-	-	-	-	F	II
	MMG_Bin_48	Acetate	II/IV	-	-	III	-	-	-	-	+	-	-	+	-	F	II
	MMG_Bin_93	Acetate & ethanol 2/3	IV/VII	-	-	III	-	-	Nitrite (pr)	N_2 (pr)	-	-	+	+	-	F	I/II
C	MMG_Bin_17	-	II/IV/VII	-	Pu	III	+	Pr	-	-	+	+	+	+	+	F	-
I	MMG_Bin_15	-	II/VII	-	-	-	+	-	-	-	+	+	+	-	-	F	-
	MMG_Bin_39	Acetate/ lactate	II/III/VII	-	Pu	-	+	Pr	Nitrite	N_2 (pr)	+	+	+	-	-	G	-
	MMG_Bin_6	Ethanol 2/3	II	-	Pr	-	+	Pr	Nitrite (pr)	N_2 (pr)	+	+	-	-	-	G	-
II	MMG_Bin_32	-	II/IV	-	Pu	III	+	Pr	-	-	+	-	-	-	-	-	-
	MMG_Bin_59	-	II/III/IV	-	-	III	+	Pr	-	-	+	-	-	-	-	-	-
III	MMG_Bin_47	Ethanol 2/3	IV	-	Pr	III	+	Pr	-	-	-	-	-	-	-	-	-
V	MMG_Bin_16	Acetate	III/IV/VII	-	-	III	-	-	Nitrite (pr)	-	-	-	+	-	-	F	-

VI	MMG_Bin_51	Lactate	IV	-	-	III	-	-	-	-	+	-	-	-	-	-	-
	MMG_Bin_85	-	II/IV	-	-	III	-	-	-	-	+	-	-	-	-	-	-
	MMG_Bin_41	-	IV	-	-	III	-	-	-	-	+	-	-	-	-	-	-
	MMG_Bin_64	Acetate	IV	-	-	III	-	-	-	-	+	-	-	-	-	-	-
	MMG_Bin_71	Acetate/ Lactate	II	-	-	-	-	-	-	-	+	-	-	-	-	-	-
	MMG_Bin_76	Lactate & ethanol 2/3	II/IV	II/III	Pr	III	-	-	-	-	+	-	-	+	-	F	-
	MMG_Bin_13	Acetate/ lactate	-	-	-	-	-	-	-	-	+	-	-	-	-	-	II
	MMG_Bin_87	Propionate 5/7	II/IV	-	-	III	-	Pr	-	-	+	-	-	+	-	-	-
	MMG_Bin_94	Acetate/ lactate	-	-	-	-	-	-	-	-	+	-	-	-	-	-	-
VII	MMG_Bin_55	Lactate	II	-	-	-	-	-	-	-	-	+	-	-	-	-	-
	MMG_Bin_90	Lactate	II	-	-	-	-	-	-	-	-	+	-	-	-	-	-
VIII	MMG_Bin_23	Ethanol 2/3	IV	-	-	III	-	-	-	-	-	-	-	-	-	-	-
	MMG_Bin_77	-	II/IV	-	-	III	-	-	-	-	-	-	-	-	-	-	-
	MMG_Bin_95	-	II/IV	-	-	III	-	-	-	-	-	-	-	-	-	-	-
Un-grouped	MMG_Bin_14	-	II/III	-	-	-	-	-	-	-	-	-	-	-	-	-	-
	MMG_Bin_26	-	-	-	-	-	-	-	-	-	-	-	-	-	-	-	-
	MMG_Bin_46	Lactate	III	-	-	-	-	-	-	-	-	-	-	-	-	-	-
	MMG_Bin_57	-	-	-	-	-	-	-	-	-	-	-	-	-	-	-	-
	MMG_Bin_5	-	II	-	-	-	-	-	-	-	-	-	-	-	-	-	-
	MMG_Bin_62	Lactate	-	-	-	-	-	-	-	-	-	-	-	-	-	-	-
	MMG_Bin_67	-	II	-	-	-	-	-	-	-	-	-	-	-	-	-	-
	MMG_Bin_80	-	-	-	-	-	-	-	-	-	-	-	-	-	-	-	-
	MMG_Bin_96	-		-	-	-	-	-	-	-	-	-	-	-	-	-	-
	MMG_Bin_104	-	-	-	-	-	-	-	-	-	+	-	-	-	-	F	-
**Old Saline Rock**																	
A	OSR_Bin_1	-	II/VII	-	Pr	-	+	Pr	-	-	-	+	+	+	+	F	II
B	OSR_Bin_39	Acetate & ethanol 2/3	II/IV	-	-	III	-	-	-	-	+	-	-	+	-	F	II
C	OSR_Bin_45	-	II/VII	-	Pr	-	+	-	Nitrite	-	+	+	+	+	+	F	-
I	OSR_Bin_21	-	II/VII	-	-	-	+	-	Nitrite (pr)	N_2O (Pr)		+	+	-	-	F	-
	OSR_Bin_36	Acetate & ethanol 2/3	II/VII	II/III	-	-	+	-	Nitrite (pr)	-	+	+	+	-	+	F	-
II	OSR_Bin_29	Acetate & propionate (5/7) & ethanol 2/3	II/VII	-	Pr	-	+	Pr	Ammonia (pr)	-	+	-	+	+	-	-	-
III	OSR_Bin_28	Acetate	II/IV/VII	-	-	III	-	Pr	Nitrite (pr)	-	-	-	+	-	-	F	-

	OSR_Bin_6	Propionate 6/7	II/IV	-	-	III	-	Pr	Nitrite (pr)	-	-	-	-	+	-	-	-
IV	OSR_Bin_13	-	II/IV	-	-	III	-	-	-	-	+	-	-	-	-	F	-
	OSR_Bin_20	Propionate 5/7	II/IV	-	-	III	-	Pr	-	-	-	-	-	+	-	F	-
Un-grouped	OSR_Bin_0	Ethanol 2/3	II	-	Pu	-	-	-	-	-	-	-	-	-	-	-	-
**Old Saline Glass**																	
B	OSG_Bin_9	Acetate & ethanol 2/3	II/IV	-	-	III	-	-	-	-	+	-	-	+	-	F	II
C	OSG_Bin_16	-	II/VII	-	-	-	+	-	-	-	+	+	+	+	+	F	-
	OSG_Bin_23	-	II	-	Pu	-	-	-	Nitrite	-	+	-	-	+	+	F	-
	OSG_Bin_24	-	II/VII	-	Pr	-	+	-	Nitrite	-	+	+	+	+	+	F	-
I	OSG_Bin_4	Acetate & ethanol 2/3	II/VII	II/III	-	-	+	-	Nitrite (pr)	-	+	+	+	-	+	F	-
II	OSG_Bin_35	Acetate & propionate (5/7) & ethanol 2/3	II/VII	-	Pu	-	+	Pr	Ammonia (pr)	-	+	-	+	+	-	F	-
III	OSG_Bin_7	Acetate	II/IV/VII	-	-	III	-	Pr	Nitrite (pr)	-	-	-	+	-	-	F	-
IV	OSG_Bin_15	-	II/IV	-	-	III	-	-	-	-	+	-	-	-	-	F	-
	OSG_Bin_2	Propionate 5/7	II/IV	-	-	III	-	Pr	-	-	+	-	-	+	-	F	-

[a] Fermentation of pyruvate to either acetate, lactate, ethanol, or propionate. Putative products have been shown where homologs of all genes in the pathway to acetate or lactate have been identified, two out of three genes for the pathway to ethanol, and five out of seven genes for the pathway to propionate.
[b] Hydrogen production via pathways I to VII.
[c] Ethanol degradation pathways I to IV.
[d] Formate oxidation to CO_2.
[e] Hydrogen oxidation pathway. The Roman numeral 'III' denotes the presence of genes for the anaerobic, NADP dependent hydrogen oxidation. This enzyme is reversible and it is possible that it produces hydrogen.
[f] Presence of the key *dsv* gene in sulfate reduction encoding dissimilatory sulfite reductase (no *dsrAB* genes were detected).
[g] Sulfur reduction to hydrogen sulfide by *psrA* via polysulfide
[h] Dissimilatory nitrate reduction has the potential end products of either nitrite or ammonia.
[i] Presence of gene homologs for nitrate reduction to nitrous oxide or all steps of nitrate reduction to nitrogen gas (denitrification).
[j] Rnf complex based on the presence of at least four of the *rnfABCDEG* genes
[k] Presence of *cbbLMS* encoding the key enzyme, ribulose bisphosphate carboxylase (RubisCO).
[l] Presence of genes for both methyl-accepting chemotaxis protein (MCP) and chemotaxis
[m] Flagella assembly based on the presence of at least twenty of *flgLKDGHIBCE*, *fliDCEFGMNHIOPQR*, *flhAB*, and all genes for the motor.
[n] Extracellular polymeric substances (EPS) production from glucose (G) or fructose (F).
[o] EPS secretion system. The Roman numeral 'I' denotes type I secretion system by *hlyBD* and *tolC*; 'II' denotes the Sec dependent type II secretion system by *epsDEFLM* , *epsDEFL*, or *epsDEFM*.
[p] '-'designates that gene homologs for the pathway/system were not identified.
[q] Pu, putative enzyme.
[r] Pr, precursor enzyme.
[s] '+'designates that gene homologs for the pathway/system were identified.
